# Silk-Nano-Fibroin Aerogels: A Bio-Derived, Amine-Rich Platform for Rapid and Reversible CO_2_ Capture

**DOI:** 10.1021/acsami.5c21809

**Published:** 2026-02-04

**Authors:** Md Sariful Sheikh, Lijie Guo, Qiyuan Chen, Bu Wang

**Affiliations:** 1 Department of Civil and Environmental Engineering, University of Wisconsin−Madison, Madison, Wisconsin 53706-1314, United States; 2 Department of Mining Engineering, Beijing General Research Institute of Mining & Metallurgy, Beijing 100160, China; 3 Department of Materials Science and Engineering, University of Wisconsin−Madison, Madison, Wisconsin 53706-1314, United States

**Keywords:** carbon capture, amino acid, silk-nano-fibroin, aerogel, biomaterial

## Abstract

Despite growing interest in biobased materials, rapid, low-temperature CO_2_ capture using amine-rich natural sorbents has received limited attention. Various porous solid sorbents have drawn significant research interest as promising carbon capture materials. However, high synthesis cost, limited CO_2_ adsorption capacity, sluggish adsorption–desorption kinetics, high sorbent regeneration temperature, and poor operational stability remain major challenges for their practical implementation. Here, we present silk-nanofibroin aerogels derived from natural mulberry silk as a sustainable, amine-rich, and porous solid-support-free sorbent platform for energy-efficient CO_2_ capture. The aerogels exhibit a CO_2_ adsorption capacity competitive with state-of-the-art amino acid and amino acid ionic liquid-based solid sorbents. Thermogravimetric analysis confirms high thermal stability up to ∼250 °C—substantially higher than that of conventional amine sorbents—while complete sorbent regeneration occurs at only 60 °C. Furthermore, the silk-nanofibroin aerogels demonstrate rapid adsorption–desorption kinetics, excellent multicycle stability, and full retention of CO_2_ adsorption capacity under humid conditions. Spectroscopic analyses (XPS, FTIR, Raman, and solid-state ^13^C NMR) confirm reversible CO_2_ chemisorption through intrinsic amine sites at the silk-fibroin surface. Overall, this work establishes silk-nanofibroin aerogels as a sustainable and low-cost route toward energy-efficient CO_2_ capture.

## Graphical abstract

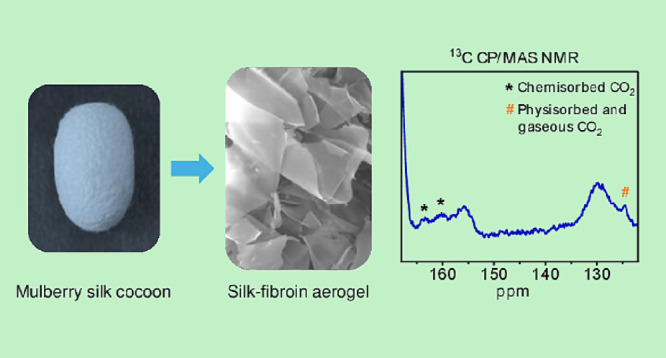

## 1 Introduction

Carbon capture, storage, and utilization are essential strategies for managing anthropogenic CO_2_ emissions and mitigating the adverse effects of ever-increasing atmospheric CO_2_ levels.^1–3^ Aqueous amine-based solvents have been practically used for CO_2_ capture for several decades,^4^ but their large-scale industrial deployment is limited by poor thermal stability (amine degradation at 100–120 °C), high regeneration temperature (≥120 °C), vaporization loss of amines due to their high vapor pressure, corrosion of the process equipment, and the adverse environmental impact of amine production and fugitive amines.^4–7^

Amino acid–based CO_2_ absorbents have drawn increasing research attention due to their amine-like CO_2_ sorption behavior, promising CO_2_ adsorption capacity, high thermal stability, nonvolatility, and eco-friendliness.^6,8–10^ However, amino acid solution technology, like the traditional aqueous amine solvents, still has several disadvantages. The high viscosity of aqueous solvents impedes CO_2_ sorption and desorption kinetics, restricting the practically achievable sorption capacity.^11^ Additionally, the regeneration of spent aqueous solutions also demands intensive heat energy due to the high heat capacity of water, resulting in high operating costs and process-related emissions.^12,13^ Alternatively, amino acid–based ionic liquids (AAILs) have demonstrated higher CO_2_ adsorption capacity compared to pure amino acids.^10,14^ Nonetheless, AAILs also face challenges such as inadequate adsorption capacity, high synthesis costs, and increased viscosity, restricting their viability for carbon capture.

To overcome these challenges, porous solid CO_2_ sorbents have emerged as an energy-efficient alternative to aqueous solvents.^1,2,4,15–17^ With a large surface area, a nanoporous sorbent can significantly reduce the sorbent regeneration energy cost due to the absence of water. Their highly porous structures facilitate rapid adsorption and desorption kinetics, overcoming the viscosity and contact area limitations of aqueous solvents. Additionally, solid sorbents can be regenerated through temperature or pressure swings, making them adaptable to various situations. A wide range of materials—including amine- and amino acid-functionalized porous structures such as silica, carbon-based materials, zeolites, metal–organic frameworks (MOFs), covalent organic frameworks (COFs), polymers, and composites—have been explored as potential sorbents.^1,18–21^ However, challenges remain in designing optimal sorbents for large-scale implementation. High-surface-area COFs and MOFs, despite their high adsorption capacity in powder form, suffer from expensive and complex synthesis processes and significant performance loss when compressed into structured sorbents.^22–26^ Carbon-based materials like graphene, carbon nanofiber, and carbon nanotubes demonstrated promising CO_2_ capture ability, but their viability is limited due to the high cost of synthesis or poor long-term stability.^27,28^ Zeolites exhibit high CO_2_ adsorption capacity but require regeneration temperatures exceeding 100 °C, leading to excessive energy consumption.^29,30^

As an alternative solid sorbent, amino acid or AAILs grafted or impregnated porous solid supports like silica gel, MOFs, COFs, and polymers have been explored and shown high CO_2_ adsorption capacities.^31–34^ Although promising, the solid amino acid/AAIL-based sorbents need further improvements in overall synthesis cost, CO_2_ adsorption capacity, desorption kinetics, and multicycle stability.^32,35,36^ Issues such as the synthesis of high-specific-surface-area solid support materials, pore blocking during amino acid grafting/impregnation, insufficient amino acid loading, and the collapse of porous support materials during operation all impact overall performance, limiting the practical implementation of these sorbents.^31,32,37,38^

Rapid low-temperature CO_2_ adsorption and desorption using bioderived, amine-rich aerogels remain underexplored because existing amino acid or amine-based sorbents typically rely on solid support materials and require high-temperature regeneration. Here, we demonstrate silk-nanofibroin aerogels that enable fast and fully reversible CO_2_ uptake and release through intrinsic amine sites, namely, surface-accessible pendant amine functionalities exposed during silk fibroin reconstruction, and interconnected mesoporous channels. This design overcomes the viscosity, pore-blocking, and energy barriers typical of supported amino-acid systems, offering a sustainable pathway toward energy-efficient CO_2_ capture using a solid sorbent.

In this work, we synthesized solid support-free silk-nanoparticles (SNPs) and silk-nanofibroin aerogels from natural mulberry silk cocoon and evaluated their CO_2_ capture performance for the first time. The silk-fibroin protein is a natural blend of amino acids, as represented in Figure S1­(a), comprising glycine (45.9%), alanine (30.30%), serine (12.1%), tyrosine (5.3%), valine (1.8%), threonine (0.9%), and other amino acids (3.7%).^39^ Silk has been widely used in diverse applications due to its low cost, biodegradability, eco-friendliness, and intrinsic chemical stability, motivating us to explore its potential as a CO_2_ sorbent.^40–42^ In addition, silk possesses unique properties like being lightweight, thermally stable, and inherently hydrophobic, which are particularly beneficial for CO_2_ capture applications.^43,44^

The resulting freeze-dried silk-nanofibroin aerogels exhibit competitive CO_2_ adsorption capacity relative to state-of-the-art amino acid and AAIL-based solid sorbents. The aerogels show excellent thermal stability (up to ∼250 °C by thermogravimetric analysis, TGA), which is much higher than that of conventional amines. Kinetic studies revealed rapid adsorption–desorption behavior and complete regeneration at only 60 °C, underscoring their potential for energy-efficient, low-temperature CO_2_ capture. The aerogels also demonstrated excellent multicycle stability and retained full adsorption capacity under humid conditions. Additionally, the spent silk-based sorbents can be naturally degraded or recycled without releasing harmful chemicals. Overall, this work establishes silk-nanofibroin aerogels as a sustainable, amine-rich, support-free sorbent platform that enables rapid, fully reversible CO_2_ capture through intrinsic amine sites and interconnected mesoporous channels.

## 2 Experimental Section

### 2.1 Synthesis of Silk-Nanofibroin Aerogel from Raw Silk Cocoon

Silk-nanofibroin aerogel was prepared by the freeze-drying of the aqueous silk-fibroin solution and hydrogel. A schematic of the silk-nanofibroin aerogel preparation process is demonstrated in Figure 1. At first, mulberry silk cocoons were cut into small pieces, boiled in a 0.05 M Na_2_CO_3_ aqueous solution for 30 min, and washed with cold DI water (i). The degumming of silk using 0.05 M Na_2_CO_3_ solution was repeated one more time. After removing the outer sericin layer by boiling, the silk was washed several times with cold water, and the degummed silk was air-dried. The dried silk-fibroin was dissolved in a warm aqueous 9.3 M LiBr solution at 65 °C and stirred for 6 h (ii).^45^ Finally, the silk-fibroin solution was dialyzed at 4 °C in a cellulose tube (molecular weight cutoff ≈ 3.5 kDa) against water to remove LiBr (iv). To determine the weight percentage of silk-fibroin in the solution, a small amount of the measured solution was dried in an oven to evaporate the water, and the weight of the silk-fibroin was calculated to determine the weight percentage of silk-fibroin in the solution. The silk-fibroin concentration in the dialyzed solution was adjusted by adding water as required. The prepared silk-fibroin solution was stored at room temperature in an airtight plastic container. The solution generally takes 2 to 6 weeks to form the silk-fibroin hydrogel. Moreover, we observed that the silk-fibroin solution forms a hydrogel only when its weight percentage exceeds 0.2%.

**Figure 1:**
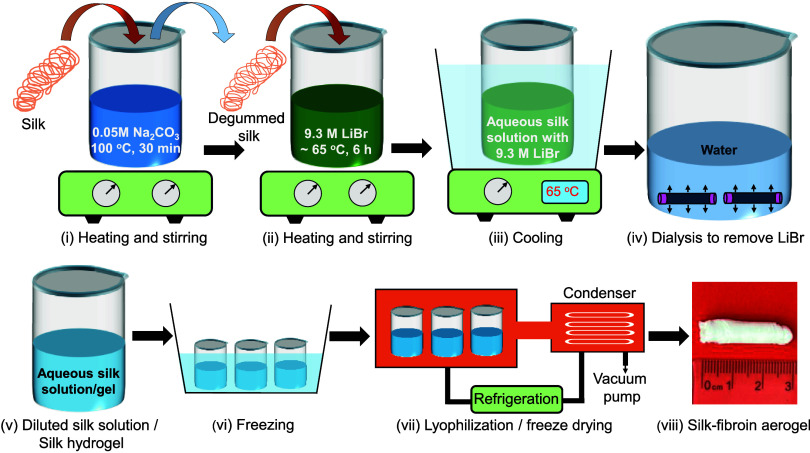
Schematic of silk-nanofibroin aerogel preparation from aqueous silk-fibroin solution and hydrogel derived from *Bombyx mori* silk.

To prepare the silk-fibroin nanostructures, the silk-fibroin solution and hydrogel were frozen using the refrigerator and liquid nitrogen at −80 °C and −196 °C, respectively (vi). Finally, the frozen silk-fibroin solution and hydrogel were lyophilized at −48 °C and 0.04 mbar to obtain the silk-nanofibroin aerogel (vii). Based on our observation, aerogel preparation from silk-fibroin solution provides a more straightforward and controllable path than aerogel preparation using silk-fibroin hydrogel, as the jellification process is time-consuming and hard to control.

The Supporting Information provides details on the SNPs synthesis (Section S1), sample characterizations (Section S2), CO_2_ capture measurements, and the methods used for the adsorption–desorption kinetics study (Section S3).

## 3 Results and Discussion

To prepare the silk-fibroin-based CO_2_ sorbent, we first attempted to prepare SNPs by partial acid hydrolysis, as represented in Figure S1­(b) and described in Section S1. Figure S2­(a–f) represents the optical images of the silk cocoon and field-effect scanning electron microscope (FESEM) images of raw silk fiber, degummed silk fiber/silk fibroin, and SNPs. The average diameter of the raw silk fiber (∼20 μm) is reduced to ∼15 μm after removing the outer sericin layer in the degummed silk. The FESEM and AFM images (Figure S3) confirm the submicron size of the silk particles. Figure 2(a) represents the high-resolution FESEM image with the roughness and porosity visible on the SNPs surface. The particle size distribution was measured using Zetasizer and presented in the inset. The plot reveals two distinct particle sizes with average diameters of 140 and 460 nm. However, the yield of silk-fibroin nanoparticles prepared by this partial acid hydrolysis method was very low, and there was significant waste of silk fibroin. Additionally, the performance of SNPs, as demonstrated later, was limited by the difficulty of controlling their porosity and specific surface area. To improve the CO_2_ capture performance of silk-fibroin, we prepared silk-nanofibroin aerogels via lyophilization of aqueous silk-fibroin solutions and hydrogels, as represented in Figure 1. The aqueous silk-fibroin solution and hydrogel were quickly frozen using liquid nitrogen at −196 °C (77K) and then freeze-dried (vacuum drying at −48 °C) to obtain the silk-nanofibroin-based aerogels. The aerogel prepared using the 0.06 wt % silk-fibroin solution, identified as sol-0.06%@77K in the rest of the manuscript, exhibits a structure composed of nanosheets and nanofibers, as shown in Figure 2(b,c). The aerogel prepared using the 0.25 wt % silk-fibroin hydrogel, identified as gel-0.25%@77K in the rest of the manuscript, represents nanosheet-like structures that are composed of very thin nanofibers, as represented in Figure 2(d–f). The specific surface area of the SNPs, sol-0.06%@77K and gel-0.25%@77K aerogels measured by the Brunauer, Emmett, and Teller (BET) method using the N_2_ adsorption isotherm at 77 K is 104.27 ± 1.89, 195.37 ± 4.97, and 232.31 ± 3.31 m^2^/g, respectively (Figure S4). The pore size distribution plot, as shown in Figure S4­(g–i) reveals the presence of micro (<2 nm) and meso-pores (>2 nm) in SNPs and gel-0.25%@77K, whereas sol-0.06%@77K has only meso-pores (>2 nm). Thus, the synthesis route strongly governs the resulting microstructure and porosity of the aerogels: rapid freeze-drying of dilute solution promotes open mesopores for faster gas diffusion, while denser hydrogel freezing preserves micro–mesoporous connectivity and higher specific surface area. This tunable pore hierarchy, derived directly from the silk-fibroin assembly pathway, underpins the promising CO_2_-adsorption capacity and rapid kinetics observed in subsequent measurements.

**Figure 2:**
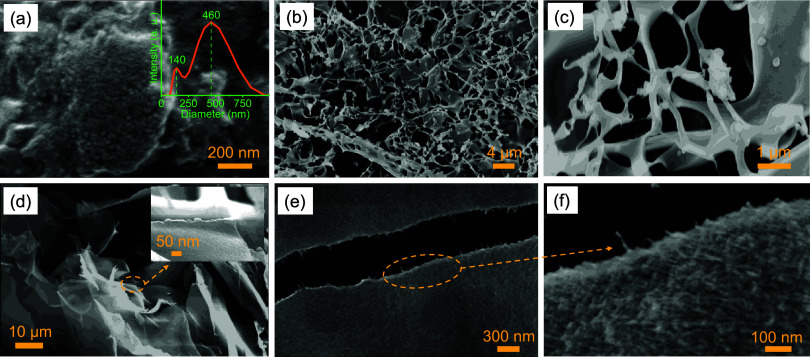
(a) FESEM image of the SNPs. Inset shows their particle size distribution. (b, c) FESEM image of the silk-nanofibroin aerogel prepared using the lyophilization of 0.06 wt % silk-fibroin solution frozen using liquid nitrogen, i.e., sol-0.06%@77K. (d–f) FESEM image of the silk-nanofibroin aerogel prepared using lyophilization of 0.25 wt % silk-fibroin hydrogel frozen using liquid nitrogen, i.e., gel-0.25%@77K.

Figure 3(a) shows the room temperature X-ray diffraction (XRD) patterns of the degummed silk, SNPs, sol-0.06%@77K, and gel-0.25%@77K aerogels. Silk fibroin has two main crystalline structures: Silk I and Silk II.^46^ The peaks at around 2θ ≈ 20.3° and 28.5° represent the presence of silk I structure, while the peak at around 2θ ≈ 24.3° belongs to the silk II structure. The SNPs and the aerogels do not show any significant change in the XRD peak positions with respect to the degummed silk, suggesting that the crystal structure of silk-fibroin remained unaffected after acid hydrolysis and lyophilization. Moreover, solid-state nuclear magnetic resonance (NMR) experiments were performed on as-prepared gel-0.25%@77K aerogel. The ^13^C cross-polarized magic-angle spinning (CP/MAS) NMR spectrum of the silk-fibroin aerogel, as shown in Figure 3(b), exhibits the significant resonances at 172.3 (Ala carbonyl carbon), 169.1 (Gly carbonyl carbon), 49.0 (Ala C_α_), 42.7 (Gly C_α_), and 19.7 (Ala C_β_) ppm, which are associated with the alanine and glycine carbonyl carbon, representing the antiparallel β-sheet crystalline (silk II) structure.^47–49^ A weaker shoulder peak at 16.4 ppm, besides the dominant Ala C_β_, indicates the presence of a silk I or distorted β-turn domains—a coexistence commonly observed in *Bombyx mori* silk-fibroin. Additional peaks are observed at 155.5, 128.5, and 114.9 ppm, which are associated with aromatic carbons of Tyr, while the peaks 63.7 and 54.5 ppm arise from the Ser C_β_ and C_α_ carbons, respectively. The peak around 91.4 ppm corresponds to a spinning sideband (SSB), which disappears at a spinning rate of 15K, as shown later. Overall, the ^13^C NMR study confirms that silk-fibroin aerogel adopts a β-sheet crystalline (Silk II) structure, with contributions from Silk I or distorted β-turn domains, consistent with our XRD observations. Figure 3(c) shows the FTIR spectra of the degummed silk, SNPs, and sol-0.06%@77K and gel-0.25%@77K, which also do not show any significant difference in the peak positions. A discussion on the observed FTIR absorption peaks is added in the Supporting Information file (Section S4). The observed absorption peaks can be attributed to amino and carboxyl groups in amino acids such as glycine and alanine. As with XRD, NMR and FTIR spectra, this suggests that the crystal structure of silk remains the same after acid hydrolysis or dissolution in salt solution. The thermal stability of the synthesized silk was examined in N_2_, CO_2_ and O_2_ gas environments using thermogravimetry, as represented in Figure S5. The study confirms that the synthesized SNPs, sol-0.06%@77K and gel-0.25%@77K, are stable up to 250 °C in inert N_2_ and CO_2_ atmospheres. However, the thermal degradation of silk starts at a slightly lower temperature in highly oxidizing conditions of pure O_2_. Overall, the TGA analysis confirms the robust thermal stability of the synthesized SNPs and aerogels. CO_2_ desorption at temperatures below 100 °C is not expected to cause material degradation, as discussed later.

**Figure 3:**
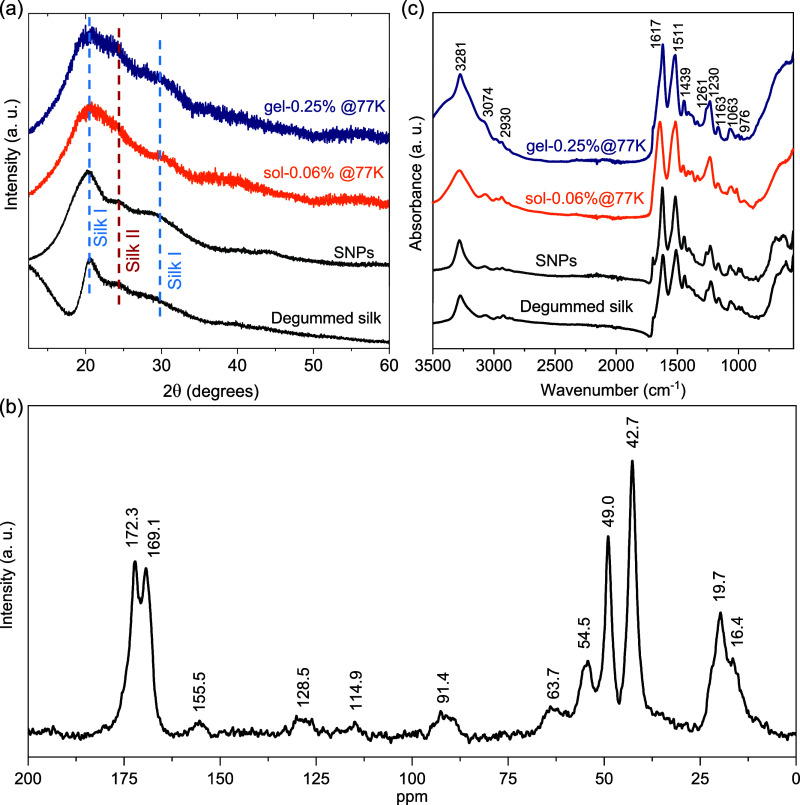
(a) Room temperature XRD patterns of the degummed silk, SNPs, sol-0.06%@77K, and gel-0.25%@77K samples. (b) ^13^C solid-state NMR spectra of as-prepared gel-0.25%@77K. Measurement was performed by cross-polarization (with continuous-wave decoupling of ^1^H). The magic angle spinning (MAS) rate was 10 kHz. (c) Fourier transform infrared (FTIR) spectra of the degummed silk, SNPs, sol-0.06%@77K, and gel-0.25%@77K samples.

The effects of freezing conditions and silk-fibroin concentration on aerogel morphology were further examined using solutions at 2, 1, 0.5, and 0.25 wt %, as shown in Figure S6. Lower solution concentration and rapid freezing in liquid nitrogen (−196 °C) produced finer nanostructures and higher specific surface area than slower freezing at −80 °C, as shown in Figure 4(a). The extremely low temperature limits molecular mobility and suppresses ice-crystal growth, yielding thin nanosheet-like frameworks instead of larger aggregated domains. Similarly, aerogels prepared from hydrogels tend to exhibit smaller structural features and higher surface areas than those from solutions of equivalent concentration, as the reduced molecular mobility in the gel phase suppresses large ice-crystal growth during freezing. However, excessive dilution reduces the synthesis yields of silk-nanofibroin aerogels and impedes gelation, limiting practical synthesis. Accordingly, three representative systems—SNPs, sol-0.06%@77K, and gel-0.25%@77K—were selected for detailed evaluation of CO_2_-capture performance. Further optimization of freezing dynamics and concentration could enable even higher surface areas and tunable pore architectures in future work.

**Figure 4:**
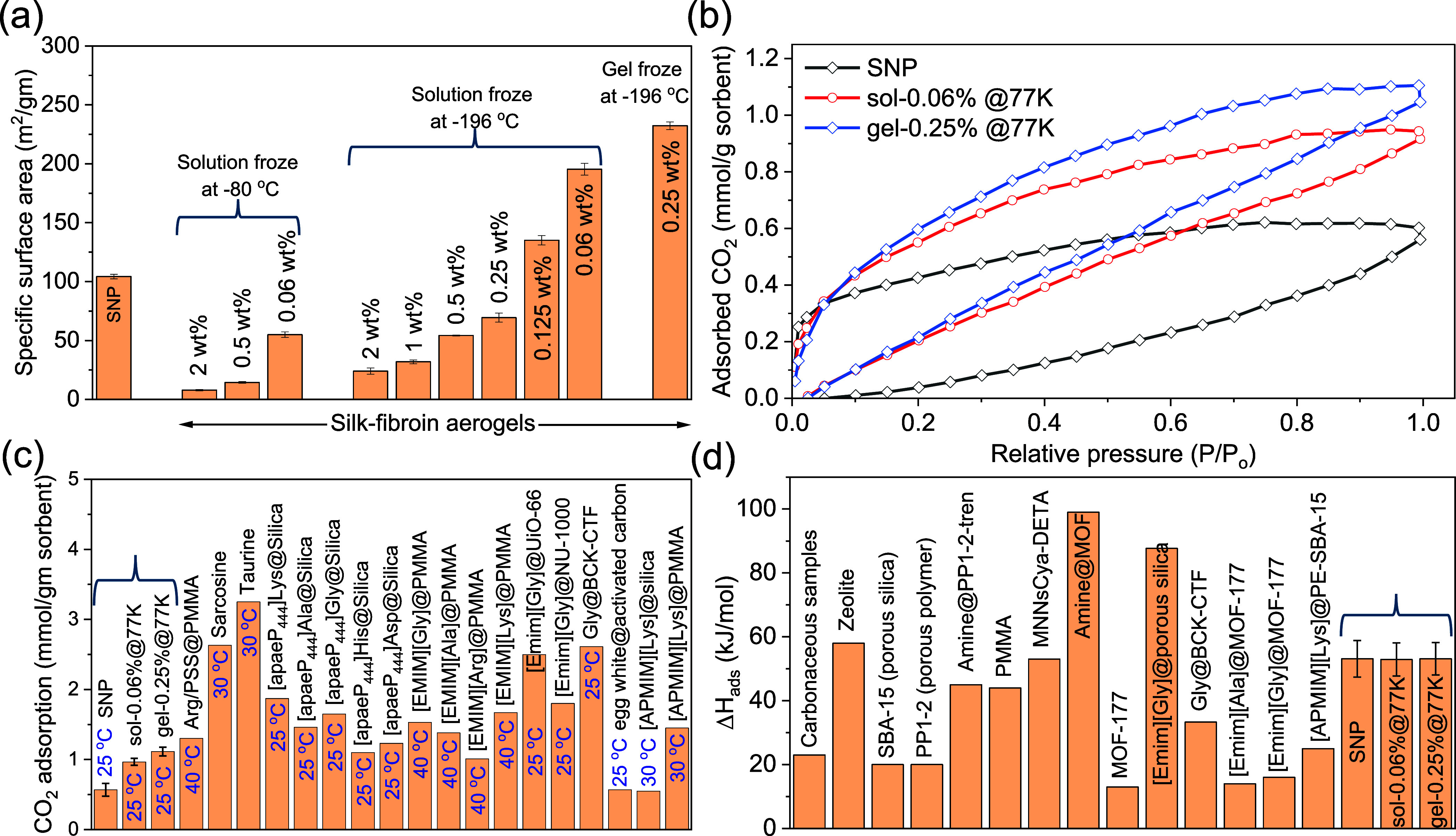
(a) Specific surface area of sorbents synthesized using partial acid hydrolysis, lyophilization of silk-fibroin solutions, and hydrogel frozen using a refrigerator (−80 °C) and liquid nitrogen (−196 °C). Data represents the average from *n* = 3 independent measurements on the same sample; error bars indicate ± standard deviation. (b) CO_2_ adsorption–desorption isotherms of SNPs, sol-0.06%@77K and gel-0.25%@77K aerogels at 25 °C. (c) The CO_2_ adsorption capacity (at 1 atm CO_2_ pressure and 25 °C) of SNPs, sol-0.06%@77K and gel-0.25%@77K, and its comparison with various amino acid–based solid sorbents (at 1 atm CO_2_ pressure and near room temperature, Table S1).^33,35,37,50–55^ Data represent the average from *n* = 3 independent measurements on the same sample; error bars indicate ± standard deviation. (d) Differential heat of adsorption (Δ*H*_ads_) of silk-fibroin-based sorbents and their comparison with various state-of-the-art sorbents like carbonaceous samples, MOFs, zeolites at 1 mmol/g CO_2_ adsorption capacity (Table S2).^2,35,53,56–62^ Average Δ*H*_ads_ values were obtained from the chemisorption region (Figure S11). Error bars represent the fitting uncertainty from the Clausius–Clapeyron analysis together with the variability among these initial Δ*H*_ads_ points.

The CO_2_ adsorption capacity of silk-fibroin-based sorbents was measured using CO_2_ adsorption–desorption isotherms at a temperature range from 5 to 25 °C, as demonstrated in Figure S7­(a–c). Figure S7­(d–f) represents the CO_2_ adsorption capacity as a function of temperature and pressure, determined from the CO_2_ adsorption–desorption isotherms. The CO_2_ adsorption capacity gradually decreases as the temperature increases and becomes poor after 25 °C. The comparison of the CO_2_ adsorption–desorption isotherms of SNPs, sol-0.06%@77K, and gel-0.25%@77K samples at 25 °C, as demonstrated in Figure 4(b), reveals relatively higher adsorption capacity of the aerogels compared to the SNPs, which could be attributed to the higher specific surface area of the aerogels as demonstrated in Figure 4(a). Figure S8 presents the magnified low-pressure region of the adsorption–desorption isotherms, showing the separation between adsorption and desorption branches and thereby illustrating the porous characteristics of the samples. The isotherm shape indicates a moderate CO_2_-amine interaction, consistent with the Δ*H*_ads_ values reported later, and supports efficient capture-release behavior under low-temperature regeneration conditions. The measured CO_2_ adsorption capacity of SNPs, sol-0.06%@77K and gel-0.25%@77K at 1 atm CO_2_ pressure and 25 °C was 0.57 ± 0.09, 0.97 ± 0.05 and 1.11 ± 0.06 mmol/g, respectively. Moreover, the adsorption capacity of synthesized sorbents is all competitive to the reported amino acid–based solid sorbents, including AAILs, many of which are synthesized explicitly with an increased number of -NH_2_ groups in the molecular chain to enhance CO_2_ adsorption, as shown in Figure 4(c) and Table S1. Most amino acid–based sorbents like taurine, sarcosine, [apaeP_444_]­[AA]@silica and [EMIM]­[AA]@PMMA, which demonstrated a higher adsorption capacity at nearly identical conditions, suffer from poor multicycle stability, with noticeable adsorption capacity drop after only a few cycles, as shown in Figures S9 and S10. On the other hand, the multicycle stability data for the sorbents Arg/PSS@PMMA, and AAIL incorporated MOFs such as [Emim]­[Gly]@UiO-66 and [Emim]­[Gly]@NU-1000 have yet not been reported. The remaining sorbent, Gly@BCK-CTF, demonstrates promising multicycle stability, but the high specific surface area of the covalent triazine framework support material (1969 m^2^/g) significantly contributes to its adsorption capacity. However, silk’s natural availability, low cost, facile synthesis, and biocompatibility, along with its promising multicycle stability (as discussed later), make free-standing silk-fibroin aerogels a stronger candidate compared to other solid support-based amino acid/AAIL sorbent materials. This suggests that silk-fibroin aerogel could be a potential candidate for CO_2_ adsorption. The high absorption capacity of the aerogels may be attributed to their large specific surface area and the presence of abundant amine group (−NH_2_) on the surface. The performance could be further increased by enhancing their specific surface area through advanced techniques, such as CO_2_ or N_2_ critical point drying, instead of the conventional freeze-drying method.

The differential adsorption enthalpy, Δ*H*_ads_, which is an important sorbent parameter to have a quantitative understanding of the thermal energy consumption required for the sorbent regeneration, was determined from the Clausius–Clapeyron relationship using experimental isotherm (adsorption) data.^61^ Figure S11­(a–c) represents the Δ*H*_ads_ dependence of the CO_2_ adsorption capacity of SNPs, sol-0.06%@77K and gel-0.25%@77K, revealing the heterogeneity of surface energy and chemical interaction between the adsorption sites as the adsorption capacity is increased.^62^ The higher Δ*H*_ads_ at lower capacity suggests chemisorption is a dominant mechanism with physisorption contributing increasingly at higher CO_2_ adsorption capacity. The sol-0.06%@77K and gel-0.25%@77K demonstrate a sudden drop in Δ*H*_ads_ value as the adsorption capacity increases above ∼1 mmol CO_2_/g, suggesting the completion of the chemisorption process by the amine groups and the start of physisorption on the remaining free space on the aerogels’ surface. However, we do not see a similar sudden drop in the Δ*H*_ads_ of SNPs, possibly due to its lower adsorption capacity. From the Δ*H*_ads_ plots of sol-0.06%@77K and gel-0.25%@77K, we speculate that these samples can chemisorb up to around ∼1 mmol/g CO_2_ by chemical interactions between the amine group of various amino acids and the CO_2_ molecule. The average Δ*H*_ads_ of SNPs was determined from the first 3 points, where chemisorption is hypothesized to be dominant. Similarly, for aerogels, Δ*H*_ads_ was estimated from the initial adsorption capacity points in Figure S11­(b,c), where chemisorption is dominant. The SNPs, sol-0.06%@77K and gel-0.25%@77K, show an average Δ*H*_ads_ value of 53.12 ± 5.74, 52.08 ± 5.19, and 53.13 ± 5.08 kJ/mol, respectively, which is comparable to the reported Δ*H*_ads_ of the state-of-the-art solid sorbents at ∼1 mmol of CO_2_/g capacity, as represented in Figure 4(d) and Table S2. The comparatively low Δ*H*_ads_ value of silk-nanofibroin-based sorbent could be the reason behind its promising CO_2_ adsorption. Moreover, it could be advantageous for fast CO_2_ desorption at lower temperatures, resulting in lower energy consumption during the cyclic adsorption–desorption process by temperature swing, as demonstrated later.

We studied the multicycle stability of the aerogels’ CO_2_ adsorption capacity using 10 cycles of adsorption–desorption isotherms at 5 °C, with the results shown in Figure 5. To study the stability of the aerogels, the samples were tested after being exposed overnight to the laboratory air at around 22 °C and a relative humidity varying between 60% and 80%. The CO_2_ gas adsorption–desorption isotherms were studied on the next day after degassing the aerogel using vacuum heating at 100 °C for 30 min. Cyclic stability was studied by monitoring the adsorption capacity at 1 atm CO_2_. The aerogel sol-0.06%@77K and gel-0.25%@77K retain their adsorption capacity after 10 cycles, showing only small fluctuations, as shown in Figure 5 **(a, b)**. The promising recyclability may be attributed to its high thermal stability and low Δ*H*_ads_, as discussed earlier. With their high sorption capacity, silk-nanofibroin aerogels show distinct characteristics compared to conventional amines, pure amino acids, and AAILs. In general, amines require higher desorption temperatures but have lower thermal degradation temperatures, resulting in poor cycling stability in practice, a major drawback of amine-based CO_2_ adsorption technologies. Figures S9 and S10 represent the CO_2_ multicycle adsorption capacity stability of some reported amino acids and AAILs-based solid sorbents, respectively. The comparison reveals that aerogels demonstrate better cyclic stability than most of these sorbents.

**Figure 5:**
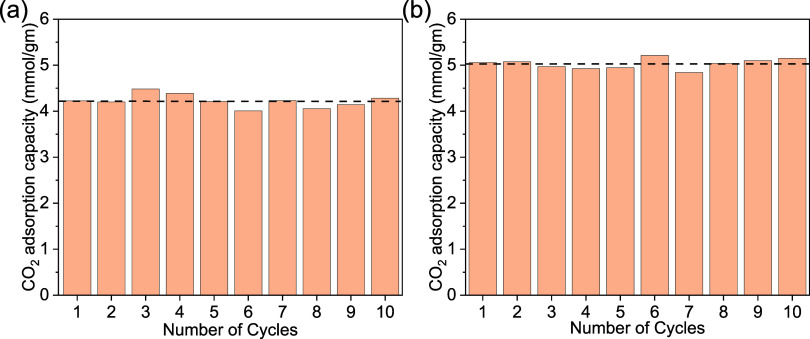
Multicycle CO_2_ adsorption stability test of (a) sol-0.06%@77K, and (b) gel-0.25%@77K.

The effect of moisture on CO_2_-adsorption performance was qualitatively examined to assess the aerogel’s stability under humid conditions. Figures 6(a) and (b) show the multicycle CO_2_ adsorption–desorption study in dry and humid ∼13.3% CO_2_-balanced N_2_ gas. To study adsorption–desorption kinetics and capacity in the presence of moisture, the aerogel gel-0.25%@77K was loaded in a U-shaped quartz tube (Figure S12). Before each cycle, the tube was placed in a 60 °C water bath to regenerate the sample under a continuous flow of 13.3% CO_2_ balanced N_2_ gas at a total flow rate of 8.3 SCCM (details in Section S3). After the regeneration, as the sample holder was transferred to a water bath at 5 °C, the CO_2_ gas concentration in the outlet of the sample holder showed an immediate decrease, suggesting CO_2_ adsorption by the aerogel. As the CO_2_ adsorbed sample is then switched to the water bath at 60 °C, the CO_2_ gas concentration in the sample holder outlet promptly increases, suggesting CO_2_ release from the sorbent. The adsorption capacity in the dry and moist conditions was compared by studying their desorption at 60 °C while sending the same dry CO_2_/N_2_ gas mixture through the sample holder tube. The average of 5 desorption peak heights, when the adsorption was performed in humid conditions (83 ± 2% relative humidity at 5 °C), showed around a 5% increase as compared to the condition when the adsorption was performed using dry ∼13.3 CO_2_-balanced N_2_ gas. However, the CO_2_ adsorption kinetics of the sorbent are slightly reduced in the presence of humidity in the gas stream, as observed in the adsorption peak height difference in Figures 6 **(a, b)**. Figure 6(c) compares the required time to complete the adsorption in dry and humid conditions, revealing slightly reduced adsorption kinetics in the presence of moisture in the adsorption gas stream. However, desorption under humid gas at 60 °C reduced the subsequent-cycle capacity, indicating that regeneration in the presence of water vapor is less efficient than under dry conditions. Overall, the silk-nanofibroin aerogel exhibits excellent moisture tolerance, retaining its CO_2_-adsorption capacity and fast adsorption–desorption kinetics under humid conditions, making it a promising candidate for CO_2_ capture.

**Figure 6:**
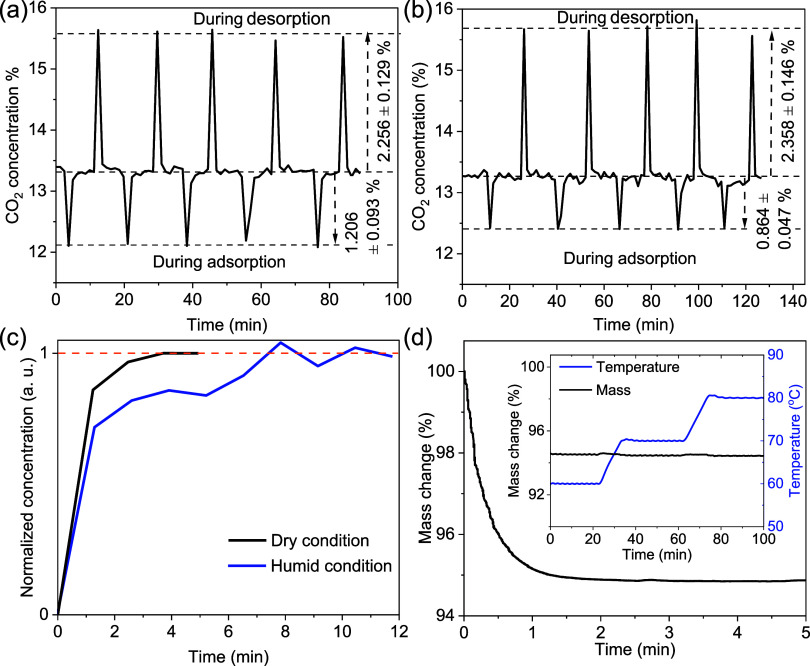
Cyclic CO_2_ adsorption and desorption at temperatures 5 and 60 °C using ∼13.3% CO_2_ balanced N_2_ gas in (a) dry and (b) humid conditions (relative humidity 83 ± 2% at 5 °C). (c) Normalized CO_2_ gas concentration at the outlet of the sorbent chamber during adsorption in dry and humid conditions. (d) CO_2_ desorption kinetics of gel-0.25%@77K at 60 °C. Inset shows the mass changes as the sample temperature increases from 60 to 80 °C stepwise.

A slow desorption rate and high temperature requirements are two major bottlenecks for implementing CO_2_ adsorption technology. Hence, we studied qualitative CO_2_ desorption kinetics using the aerogel gel-0.25%@77K. To study CO_2_ desorption kinetics, CO_2_ adsorption was first performed by exposing the aerogel to 1 atm of CO_2_ at 23 °C for 15 min. Its mass change was then measured in a 1 atm CO_2_ environment at 60 °C using TGA. Figure 6(d) presents the CO_2_ desorption kinetics of gel-0.25%@77K sample at 60 °C. The TGA shows a nearly 5% mass drop after regeneration, which is quantitatively consistent with a CO_2_ capture capacity of ∼1.11 mmol CO_2_/g aerogel (∼0.046 g CO_2_/g) at 25 °C, as observed in a CO_2_ adsorpti on–desorption study. The sample releases all adsorbed CO_2_ within 3 min, demonstrating the very fast sorbent regeneration, which can be attributed to its low heat of adsorption. We also noticed that increasing the sample temperature from 60 to 80 °C resulted in no noticeable mass change, as shown in Figure 6(d) inset. This suggests that sorbent regeneration was complete at 60 °C. The rapid sorbent regeneration at low temperature reveals the promising potential of self-supported silk-nanofibroin aerogel for energy-efficient CO_2_ capture.

To elucidate the CO_2_ adsorption mechanism on silk-nanofibroin aerogels, X-ray photoemission spectroscopy (XPS), Raman, FTIR, and solid-state ^13^C NMR spectroscopy analyses were performed. The XPS survey spectra of CO_2_ adsorbed aerogel before and after Ar^+^ ion sputtering (Figure S13) show a decrease in surface carbon (57.67% to 53.05 atomic %) and an increase in surface nitrogen (18.5 to 21.52 atomic %), indicating the removal of chemisorbed CO_2_ and re-exposure of surface -NH_2_ groups. The deconvolution of the core level C 1s spectra, as shown in Figure 7(a) and Figure S13 **(c, d)**, reveals a slight overall increase in the C–C peak height. The fitting parameters (Table S3) also indicate a slight increase in the C–C bond area percentage (32.79% to 33.57%). In contrast, the area percentage of the C–OH/C–N and O–C = O/N–C = O bonds decreases slightly, confirming amine-assisted CO_2_ chemisorption on the aerogel surface.

**Figure 7:**
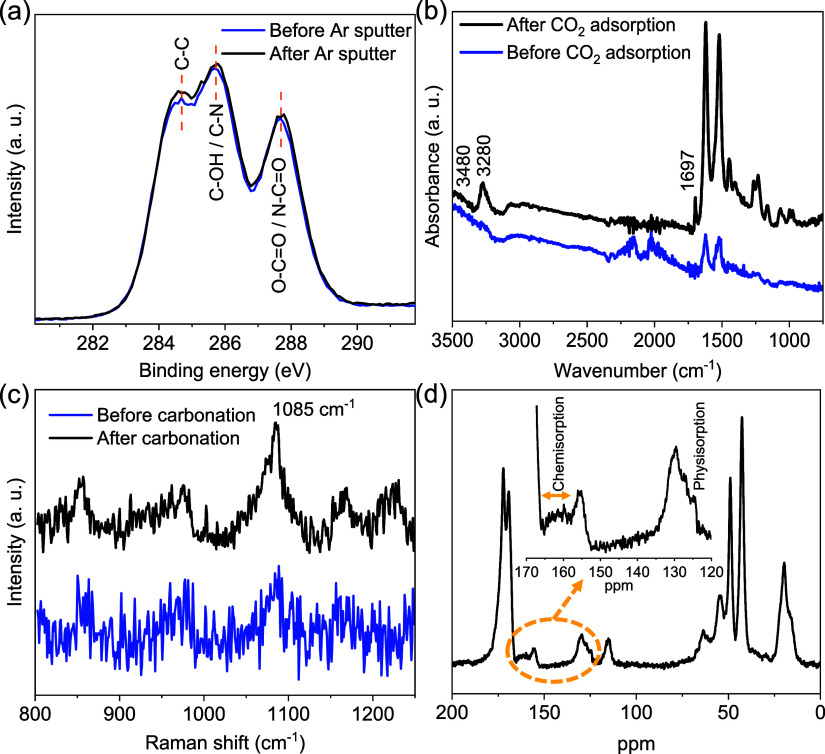
(a) Comparison of the high-resolution C 1s spectra of the CO_2_ adsorbed sorbent before and after monatomic Ar^+^ ion sputtering on the sorbent surface. (b) FTIR spectra of the sorbent before and after CO_2_ adsorption. (c) Raman spectra before and after CO_2_ adsorption were collected in N_2_ and CO_2_ environments, respectively. (d) ^13^C solid-state NMR spectra of silk-fibroin aerogel after ^13^CO_2_ adsorption at atmospheric pressure and at room temperature. NMR measurements were performed using cross-polarization (with continuous-wave decoupling of 1H) with a total of 1024 scans. The MAS rate was 15 kHz. During NMR measurement, the sample’s temperature was calculated as 22.5 ± 0.2 °C.

FTIR spectra, as shown in Figure **7­(b)**, further supported this interpretation. Upon CO_2_ exposure, a new band emerges near 1697 cm^–1^, characteristic of carbamate formation.^51^ Additionally, the secondary amine (−NH) at 3280 cm^–1^ intensified as the primary amine (−NH_2_) group transforms to the secondary amine (−NH) upon carbamate formation, as represented in eqs 1–3). The peak associated with the primary amine (−NH_2_) group in CO_2_ desorbed sample is positioned at a slightly higher wavenumber and overlapped with the broad hydroxyl group peak at 3480 cm^–1^, not clearly distinguishable. These changes signify the transformation of – NH_2_ to – NH–COO^–^/–NHCOOH species, in agreement with previous reports.^51,63,64^ Raman spectroscopy, as represented in Figure 7(c), revealed an enhanced carbonate (CO_3_^2–^) vibration at approximately 1085 cm^–1^ in the CO_2_-adsorbed sample compared to the CO_2_ desorbed controlled sample,^65^ again confirming the chemisorption of CO_2_ molecules on the silk-nanofibroin aerogel surface.

To selectively probe the adsorbed carbon atoms, ^13^CO_2_ gas was employed in solid-state ^13^C cross-polarized magic angle spinning (CP/MAS) NMR. The spectrum of the silk-fibroin aerogel after ^13^CO_2_ gas adsorption at ∼22.5 C, as shown in Figure 7(d), reveals the appearance of new resonances between 158 and 165 ppm and a sharp line at around 124.6 ppm, as compared to the as-prepared aerogel sample, as shown in Figure 3(b). The 158–165 ppm region corresponds to chemisorbed CO_2_ species (ammonium carbamate and/or carbamic acid), whereas the 124.6 ppm line arises from physisorbed and gaseous ^13^CO_2_ confined within the pores.^66–70^ Decreasing the sample temperature to around ∼7 °C makes the resonance peaks more prominent with the distinct appearance of chemisorb peaks at 160.2 and 163.5 ppm and physiosorbed/gas phase peak at 124.6 ppm, as shown in Figure S14. The coexistence of both chemisorbed and physisorbed CO_2_ is consistent with amine-functionalized silicas or MOFs.^66,67,69^ However, in addition to the nearly overlapping feature of chemisorbed resonances, the carbon-rich background of the silk-fibroin matrix makes the overall chemisorbed ^13^C peak relatively weaker than in systems where amine groups were incorporated into MOF or silica frameworks.

Collectively, XPS, FTIR, Raman, and solid-state NMR results demonstrate that CO_2_ is chemisorbed through surface amine groups of the silk-fibroin backbone, forming carbamate and carbamic-acid species. Under dry conditions, two amine groups react with one CO_2_ molecule to form an ammonium carbamate (eqs 1–2). Whereas, in the presence of moisture, one amine group reacts with one CO_2_ molecule to form a bicarbonate ion (eq 3),^71^ explaining the observed ∼5% increase in CO_2_ uptake under moisture.

$$R-{\mathrm{NH}}_{2}+{\mathrm{CO}}_{2}↔R-\mathrm{NHCOOH}(\mathrm{Carbamic}\;\mathrm{acid})$$ 1

$$R-\mathrm{NHCOOH}+R-{\mathrm{NH}}_{2}↔R-{\mathrm{NHCOO}}^{-}(\mathrm{Carbamate}\;\mathrm{ion})+R-{{\mathrm{NH}}_{3}}^{+}$$ 2

$$R-{\mathrm{NH}}_{2}+{\mathrm{CO}}_{2}+H_{2}O↔R-{{\mathrm{NH}}_{3}}^{+}+{{\mathrm{HCO}}_{3}}^{-}(\mathrm{Bicarbonate}\;\mathrm{ion})$$ 3

The intrinsic amino-acid composition of silk-nanofibroin aerogel, which is rich in glycine, alanine, serine, and tyrosine, provides abundant amine-rich binding sites responsible for the observed rapid CO_2_ capture–release kinetics. Compared with state-of-the-art pure amino acids and AAIL functionalized solid sorbents, this bioderived, free-standing silk-nanofibroin aerogel offers cost-effectiveness, biocompatibility, robust thermal and moisture stability, and cyclic adsorption–desorption stability. Together, these attributes position silk-derived aerogels as an energy-efficient and sustainable platform for low-temperature, low-energy CO_2_ capture, in which rapid adsorption–desorption kinetics and mild regeneration conditions are prioritized over maximum equilibrium uptake. Future studies may assess its long-term operational stability under flue-gas conditions, and explore optimization of the pore architecture and surface chemistry to achieve tunable Δ*H*_ads_ for enhanced high-temperature uptake, and evaluate process-level techno-economic factors to advance this natural-protein-based platform toward practical carbon-capture deployment.

## 4 Conclusions

In this study, silk-nanofibroin aerogels derived from natural mulberry silk were developed and systematically evaluated as sustainable, amine-rich solid sorbents for CO_2_ capture. The aerogel exhibits a high specific surface area and competitive CO_2_ adsorption capacity, comparable to state-of-the-art amino acid–based solid sorbents. TGA confirms excellent thermal stability up to ∼250 °C, while the material maintains full CO_2_-uptake capacity during repeated adsorption–desorption cycles and under humid conditions. Furthermore, the aerogels demonstrate rapid adsorption–desorption kinetics and complete regeneration at only 60 °C, underscoring their potential for low-energy CO_2_ capture. Overall, this work establishes silk-nanofibroin aerogels as a sustainable, amine-rich, support-free sorbent platform that enables rapid and fully reversible CO_2_ capture through surface accessible amine sites and interconnected mesoporous channels. These results highlight the promise of natural-protein-derived materials for scalable, energy-efficient carbon-capture technologies and motivate further exploration of silk-based sorbents toward practical and low-cost CO_2_-separation processes.

## Supplementary Material



## Data Availability

The Figure data and raw data, as presented in the main manuscript and the Supporting Information, are openly available in Figshare at 10.6084/m9.figshare.30460787.^72^

## Notes

Details of SNP synthesis, materials characterization methods, including XRD, FTIR, solid-state NMR, AFM, FESEM, XPS, Raman spectroscopy, and gas adsorption–desorption studies; comparative table of the CO2 adsorption capacity, Δ*H*_ads_, and XPS fitting parameters (PDF)

## References

[1] Charalambous C., Moubarak E., Schilling J., Sanchez Fernandez E., Wang J.-Y., Herraiz L., Mcilwaine F., Peh S. B., Garvin M., Jablonka K. M., Moosavi S. M., Van Herck J., Ozturk A. Y., Pourghaderi A., Song A.-Y., Mouchaham G., Serre C., Reimer J. A., Bardow A., Smit B., Garcia S. (2024). A Holistic Platform
for Accelerating Sorbent-Based Carbon Capture. Nature.

[2] Mao H., Tang J., Day G. S., Peng Y., Wang H., Xiao X., Yang Y., Jiang Y., Chen S., Halat D. M., Lund A., Lv X., Zhang W., Yang C., Lin Z., Zhou H.-C., Pines A., Cui Y., Reimer J. A. (2022). A Scalable Solid-State Nanoporous Network with Atomic-Level
Interaction Design for Carbon Dioxide Capture. Science Advances.

[3] Ragipani R., Sreenivasan K., Anex R. P., Zhai H., Wang B. (2022). Direct Air
Capture and Sequestration of CO2 by Accelerated Indirect Aqueous Mineral
Carbonation under Ambient Conditions. ACS Sustainable
Chem. Eng..

[4] D’Alessandro D. M., Smit B., Long J. R. (2010). Carbon
Dioxide Capture: Prospects
for New Materials. Angew. Chem., Int. Ed..

[5] Hack J., Maeda N., Meier D. M. (2022). Review on CO2 Capture Using Amine-Functionalized
Materials. ACS Omega.

[6] Sang
Sefidi V., Luis P. (2019). Advanced Amino Acid-Based Technologies
for CO2 Capture: A Review. Ind. Eng. Chem. Res..

[7] Zhao W., Zhang Z., Li Z., Cai N. (2013). Investigation of Thermal
Stability and Continuous CO2 Capture from Flue Gases with Supported
Amine Sorbent. Ind. Eng. Chem. Res..

[8] Hu G., Smith K. H., Wu Y., Mumford K. A., Kentish S. E., Stevens G. W. (2018). Carbon Dioxide Capture
by Solvent Absorption Using
Amino Acids: A Review. Chinese Journal of Chemical
Engineering.

[9] Ramezani R., Mazinani S., Di Felice R. (2022). State-of-the-Art
of CO2 Capture with
Amino Acid Salt Solutions. Rev. Chem. Eng..

[10] Sheikh M. S., Sui J., Wang B., Wang X. (2025). CO2 Capture, Utilization, and Storage
Using Amino Acids. Adv. Sustainable Syst..

[11] Raganati F., Miccio F., Ammendola P. (2021). Adsorption
of Carbon Dioxide for
Post-Combustion Capture: A Review. Energy Fuels.

[12] Rabensteiner M., Kinger G., Koller M., Hochenauer C. (2015). PCC Pilot
Plant Studies with Aqueous Potassium Glycinate. International Journal of Greenhouse Gas Control.

[13] Alivand M. S., Mazaheri O., Wu Y., Stevens G. W., Scholes C. A., Mumford K. A. (2019). Development of Aqueous-Based
Phase Change Amino Acid
Solvents for Energy-Efficient CO2 Capture: The Role of Antisolvent. Applied Energy.

[14] Saravanamurugan S., Kunov-Kruse A. J., Fehrmann R., Riisager A. (2014). Amine-Functionalized
Amino Acid-Based Ionic Liquids as Efficient and High-Capacity Absorbents
for CO2. ChemSusChem.

[15] Lu C., Zhang X., Chen X. (2022). Advanced Materials
and Technologies
toward Carbon Neutrality. Acc. Mater. Res..

[16] Shi X., Xiao H., Azarabadi H., Song J., Wu X., Chen X., Lackner K. S. (2020). Sorbents for the Direct Capture of
CO2 from Ambient Air. Angew. Chem., Int. Ed..

[17] Wang S., Mahurin S. M., Dai S., Jiang D. (2021). Design of Graphene/Ionic
Liquid Composites for Carbon Capture. ACS Appl.
Mater. Interfaces.

[18] Zentou H., Hoque B., Abdalla M. A., Saber A. F., Abdelaziz O. Y., Aliyu M., Alkhedhair A. M., Alabduly A. J., Abdelnaby M. M. (2025). Recent
Advances and Challenges in Solid Sorbents for CO2 Capture. Carbon Capture Science & Technology.

[19] Siegelman R. L., Kim E. J., Long J. R. (2021). Porous
Materials for Carbon Dioxide
Separations. Nat. Mater..

[20] Liu H., Zhao W., Lu H., Meng H. (2025). Encapsulated Ionic
Liquids with MOF-Driven CO2 Channels: Overcoming Kinetic Limits for
Rapid Carbon Capture. ACS Appl. Mater. Interfaces.

[21] Deeg K. S., Damasceno Borges D., Ongari D., Rampal N., Talirz L., Yakutovich A. V., Huck J. M., Smit B. (2020). In Silico Discovery
of Covalent Organic Frameworks for Carbon Capture. ACS Appl. Mater. Interfaces.

[22] Sher F., Hayward A., Guerraf A. E., Wang B., Ziani I., Hrnjić H., Boškailo E., Chupin A., Nemţanu M. R. (2024). Advanced
Metal–Organic Frameworks for Superior Carbon Capture, High-Performance
Energy Storage and Environmental Photocatalysis – a Critical
Review. J. Mater. Chem. A.

[23] Bui M., Adjiman C. S., Bardow A., Anthony E. J., Boston A., Brown S., Fennell P. S., Fuss S., Galindo A., Hackett L. A., Hallett J. P., Herzog H. J., Jackson G., Kemper J., Krevor S., Maitland G. C., Matuszewski M., Metcalfe I. S., Petit C., Puxty G., Reimer J., Reiner D. M., Rubin E. S., Scott S. A., Shah N., Smit B., Trusler J. P. M., Webley P., Wilcox J., Dowell N. M. (2018). Carbon Capture and Storage (CCS): The Way Forward. Energy Environ. Sci..

[24] Mazaj M., Logar N. Z., Žagar E., Kovačič S. (2017). A Facile Strategy
towards a Highly Accessible and Hydrostable MOF-Phase within Hybrid
polyHIPEs through in Situ Metal-Oxide Recrystallization. J. Mater. Chem. A.

[25] Zhang Z., Dai Y., Zhang S., Chen L., Gu J., Wang Y., Sun W. (2025). Porous Framework
Materials for CO2 Capture. Journal of Energy
Chemistry.

[26] Sutton A. L., Sadiq M. M., Scalzo M. T., Seeber A., Bird S. A., Konstas K., Mardel J. I. (2025). Understanding Porosity Loss in MOF
Pelletization: Intrinsic vs Extrinsic Mechanical Stability in Zn-MOF-74. ACS Omega.

[27] Ramar V., Balraj A. (2022). Critical Review on
Carbon-Based Nanomaterial for Carbon
Capture: Technical Challenges, Opportunities, and Future Perspectives. Energy Fuels.

[28] Isah M., Lawal R., Onaizi S. A. (2025). CO2 Capture
and Conversion Using
Graphene-Based Materials: A Review on Recent Progresses and Future
Outlooks. Green Chemical Engineering.

[29] Boer D. G., Langerak J., Pescarmona P. P. (2023). Zeolites as Selective Adsorbents
for CO2 Separation. ACS Appl. Energy Mater..

[30] Ganiyu S. A. (2025). Review
of Zeolitic-Based Sorbents for CO2 Capture: Insights into Functional
Modifications, Economic Feasibility and Machine Learning-Enhanced
Strategies. Chemical Engineering Journal Advances.

[31] Sun L., Gao M., Tang S. (2021). Porous Amino
Acid-Functionalized Poly­(Ionic Liquid)
Foamed with Supercritical CO2 and Its Application in CO2 Adsorption. Chemical Engineering Journal.

[32] Mohamed
Hatta N. S., Aroua M. K., Hussin F., Gew L. T. (2022). A Systematic
Review of Amino Acid-Based Adsorbents for CO2 Capture. Energies.

[33] Wang X., Akhmedov N. G., Duan Y., Luebke D., Hopkinson D., Li B. (2013). Amino Acid-Functionalized Ionic Liquid Solid Sorbents for Post-Combustion
Carbon Capture. ACS Appl. Mater. Interfaces.

[34] Uehara Y., Karami D., Mahinpey N. (2019). Amino Acid
Ionic Liquid-Modified
Mesoporous Silica Sorbents with Remaining Surfactant for CO2 Capture. Adsorption.

[35] Dong B., Wang D.-Y., Wang W.-J., Tian X.-L., Ren G. (2020). Post Synthesis
of a Glycine-Functionalized Covalent Triazine Framework with Excellent
CO2 Capture Performance. Microporous Mesoporous
Mater..

[36] Lyu H., Chen O. I.-F., Hanikel N., Hossain M. I., Flaig R. W., Pei X., Amin A., Doherty M. D., Impastato R. K., Glover T. G., Moore D. R., Yaghi O. M. (2022). Carbon Dioxide Capture
Chemistry of Amino Acid Functionalized Metal–Organic Frameworks
in Humid Flue Gas. J. Am. Chem. Soc..

[37] Jiang B., Wang X., Gray M. L., Duan Y., Luebke D., Li B. (2013). Development of Amino Acid and Amino Acid-Complex Based Solid Sorbents
for CO2 Capture. Applied Energy.

[38] Huang Z., Karami D., Mahinpey N. (2021). Study on the
Efficiency of Multiple
Amino Groups in Ionic Liquids on Their Sorbents Performance for Low-Temperature
CO2 Capture. Chem. Eng. Res. Des..

[39] Babu, K. M. 11 - Silk Fibres – Structure, Properties and Applications. In Handbook of Natural Fibres, (Second ed.); Kozłowski, R. M. , Mackiewicz-Talarczyk, M. , Eds.; Woodhead Publishing Series in Textiles; Woodhead Publishing: 2020; pp 385–416. 10.1016/B978-0-12-818398-4.00013-X.

[40] Wigham C., Fink T. D., Sorci M., O’Reilly P., Park S., Kim J., Varude V. R., Zha R. H. (2024). Phosphate-Driven
Interfacial Self-Assembly of Silk Fibroin for Continuous Noncovalent
Growth of Nanothin Defect-Free Coatings. ACS
Appl. Mater. Interfaces.

[41] Oral C. B., Su E., Okay O. (2024). Silk Fibroin-Based
Multiple-Shape-Memory Organohydrogels. ACS Appl.
Mater. Interfaces.

[42] Tan X. H., Liu L., Mitryashkin A., Wang Y., Goh J. C. H. (2022). Silk Fibroin
as a Bioink – A Thematic Review of Functionalization Strategies
for Bioprinting Applications. ACS Biomater.
Sci. Eng..

[43] Tao H., Kaplan D. L., Omenetto F. G. (2012). Silk Materials
– A Road to
Sustainable High Technology. Adv. Mater..

[44] Zhou Z., Zhang S., Cao Y., Marelli B., Xia X., Tao T. H. (2018). Engineering the Future of Silk Materials through Advanced
Manufacturing. Adv. Mater..

[45] Rockwood D. N., Preda R. C., Yücel T., Wang X., Lovett M. L., Kaplan D. L. (2011). Materials Fabrication
from Bombyx Mori Silk Fibroin. Nat. Protoc.

[46] Xing L., Wang Y., Cheng J., Chen G., Xing T. (2023). Robust and
Flexible Smart Silk/PEDOT Conductive Fibers as Wearable Sensor for
Personal Health Management and Information Transmission. Int. J. Biol. Macromol..

[47] Asakura T., Yao J., Yamane T., Umemura K., Ulrich A. S. (2002). Heterogeneous Structure
of Silk Fibers from Bombyx Mori Resolved by 13C Solid-State NMR Spectroscopy. J. Am. Chem. Soc..

[48] Nakazawa C. T., Higuchi A., Asano A., Kameda T., Aytemiz D., Nakazawa Y. (2017). Solid-State NMR Studies
for the Development of Non-Woven
Biomaterials Based on Silk Fibroin and Polyurethane. Polym. J..

[49] Pham D. T., Saelim N., Tiyaboonchai W. (2018). Crosslinked Fibroin Nanoparticles
Using EDC or PEI for Drug Delivery: Physicochemical Properties, Crystallinity
and Structure. J. Mater. Sci..

[50] Chatterjee S., Rayalu S., Kolev S. D., Krupadam R. J. (2016). Adsorption of Carbon
Dioxide on Naturally Occurring Solid Amino Acids. Journal of Environmental Chemical Engineering.

[51] Ren J., Wu L., Li B.-G. (2012). Preparation
and CO2 Sorption/Desorption of N-(3-Aminopropyl)­Aminoethyl
Tributylphosphonium Amino Acid Salt Ionic Liquids Supported into Porous
Silica Particles. Ind. Eng. Chem. Res..

[52] Balsamo M., Erto A., Lancia A., Totarella G., Montagnaro F., Turco R. (2018). Post-Combustion CO2
Capture: On the
Potentiality of Amino Acid Ionic Liquid as Modifying Agent of Mesoporous
Solids. Fuel.

[53] Mohamed
Hatta N. S., Hussin F., Gew L. T., Aroua M. K. (2023). Enhancing
Surface Functionalization of Activated Carbon Using Amino Acids from
Natural Source for CO2 Capture. Sep. Purif.
Technol..

[54] Huang Z., Mohamedali M., Karami D., Mahinpey N. (2022). Evaluation of Supported
Multi-Functionalized Amino Acid Ionic Liquid-Based Sorbents for Low
Temperature CO2 Capture. Fuel.

[55] Xia X., Hu G., Li W., Li S. (2019). Understanding Reduced CO2 Uptake
of Ionic Liquid/Metal–Organic Framework (IL/MOF) Composites. ACS Appl. Nano Mater..

[56] Xu C., Bacsik Z., Hedin N. (2015). Adsorption
of CO2 on a Micro-/Mesoporous
Polyimine Modified with Tris­(2-Aminoethyl)­Amine. J. Mater. Chem. A.

[57] Philip F. A., Henni A. (2023). Incorporation of Amino
Acid-Functionalized Ionic Liquids into Highly
Porous MOF-177 to Improve the Post-Combustion CO2 Capture Capacity. Molecules.

[58] Fan X., Zhang L., Zhang G., Shu Z., Shi J. (2013). Chitosan Derived
Nitrogen-Doped Microporous Carbons for High Performance CO2 Capture. Carbon.

[59] Bae T.-H., Hudson M. R., Mason J. A., Queen W. L., Dutton J. J., Sumida K., Micklash K. J., Kaye S. S., Brown C. M., Long J. R. (2013). Evaluation of Cation-Exchanged Zeolite
Adsorbents for
Post-Combustion Carbon Dioxide Capture. Energy
Environ. Sci..

[60] Mohamedali M., Ibrahim H., Henni A. (2020). Imidazolium Based Ionic Liquids Confined
into Mesoporous Silica MCM-41 and SBA-15 for Carbon Dioxide Capture. Microporous Mesoporous Mater..

[61] Kim E. J., Siegelman R. L., Jiang H. Z. H., Forse A. C., Lee J.-H., Martell J. D., Milner P. J., Falkowski J. M., Neaton J. B., Reimer J. A., Weston S. C., Long J. R. (2020). Cooperative
Carbon Capture and Steam Regeneration with Tetraamine-Appended Metal–Organic
Frameworks. Science.

[62] Sheshkovas A. Z., Veselovskaya J. V., Rogov V. A., Kozlov D. V. (2022). Thermochemical Study
of CO2 Capture by Mesoporous Silica Gel Loaded with the Amino Acid
Ionic Liquid 1-Ethyl-3-Methylimidazolium Glycinate. Microporous Mesoporous Mater..

[63] Sistla Y. S., Khanna A. (2015). CO2 Absorption Studies
in Amino Acid-Anion Based Ionic
Liquids. Chemical Engineering Journal.

[64] Wu J., Yang Z., Xie J., Zhu P., Wei J., Jin R., Yang H. (2023). Porous Polymer Supported
Amino Functionalized Ionic
Liquid for Effective CO2 Capture. Langmuir.

[65] Hallenbeck A. P., Egbebi A., Resnik K. P., Hopkinson D., Anna S. L., Kitchin J. R. (2015). Comparative Microfluidic Screening
of Amino Acid Salt Solutions for Post-Combustion CO2 Capture. International Journal of Greenhouse Gas Control.

[66] Fonseca R., Vieira R., Sardo M., Marin-Montesinos I., Mafra L. (2022). Exploring Molecular Dynamics of Adsorbed
CO2 Species in Amine-Modified
Porous Silica by Solid-State NMR Relaxation. J. Phys. Chem. C.

[67] Forse A. C., Milner P. J., Lee J.-H., Redfearn H. N., Oktawiec J., Siegelman R. L., Martell J. D., Dinakar B., Zasada L. B., Gonzalez M. I., Neaton J. B., Long J. R., Reimer J. A. (2018). Elucidating
CO2 Chemisorption in Diamine-Appended Metal–Organic Frameworks. J. Am. Chem. Soc..

[68] Jasil M., Thomas B. (2025). Solid State NMR for
Mechanistic Exploration of CO2
Adsorption on Amine-Based Silica Adsorbents. ACS Omega.

[69] Liao P.-Q., Chen X.-W., Liu S.-Y., Li X.-Y., Xu Y.-T., Tang M., Rui Z., Ji H., Zhang J.-P., Chen X.-M. (2016). Putting an Ultrahigh Concentration
of Amine Groups
into a Metal–Organic Framework for CO 2 Capture at Low Pressures. Chem. Sci..

[70] Li Y., Duan X., Song W., Ma L., Jow J. (2021). Reaction Mechanisms
of Carbon Dioxide Capture by Amino Acid Salt and Desorption by Heat
or Mineralization. Chemical Engineering Journal.

[71] Uehara Y., Karami D., Mahinpey N. (2018). Roles of Cation
and Anion of Amino
Acid Anion-Functionalized Ionic Liquids Immobilized into a Porous
Support for CO2 Capture. Energy Fuels.

[72] Data for Manuscript Entitled “Silk-Nano-Fibroin Aerogels: A Bio-Derived, Amine-Rich Platform for Rapid and Reversible CO2 Capture”; Figshare: 2025. 10.6084/m9.figshare.30460787. PMC12926954 41636618

