# Supplementary material for: Silk-Nano-Fibroin Aerogels: A Bio-Derived, Amine-Rich Platform for Rapid and Reversible CO2 Capture
Source: ACS Appl Mater Interfaces. 2026 Feb 4;18(6):9778–88. doi: 10.1021/acsami.5c21809 (PMC12926954; doi:10.1021/acsami.5c21809)
Supplement: Supplementary file 1 [file am5c21809_si_001.pdf]

Supporting Information File

Silk-Nano-Fibroin Aerogels: A Bio-Derived, Amine-Rich Platform for Rapid and Reversible CO_2 Capture

Md Sariful Sheikh,[1] Lijie Guo,[2] Qiyuan Chen,[3] Bu Wang[1,]*

[1] Department of Civil and Environmental Engineering, University of Wisconsin−Madison, Madison, Wisconsin 53706-1314, United States
[2] Department of Mining Engineering, Beijing General Research Institute of Mining & Metallurgy, Beijing 100160, China
[3] Department of Materials Science and Engineering, University of Wisconsin−Madison, Madison, Wisconsin 53706-1314, United States

*Email: bu.wang@wisc.edu

Table of contents

❖ Supplementary Figures:

Figure S1: (a) Percentage of various amino acids in the silk-fibroin[1]. (b) A schematic of the porous silk-nanoparticles (SNPs) synthesis from mulberry silk cocoon.

Figure S2: (a) Optical image of the mulberry silk cocoon. (b) Field effect scanning electron microscope (FESEM) image of the raw silk fiber. (c) FESEM image of the degummed silk fiber. (d, e, f) FESEM image of the SNPs at various resolutions.

Figure S3: AFM (a, b, c) topography image; simultaneously acquired (d, e, f) amplitude; and (g, h, i) phase image of the SNPs.

Figure S4: N_2 adsorption-desorption isotherm of (a) SNPs, (b) sol-0.06%@77K, and (c) gel-0.25%@77K aerogels at 77 K. Specific surface area analysis of (d) SNPs, (e) sol-0.06%@77K, and (f) gel-0.25%@77K using the Brunauer–Emmett–Teller (BET) method. Pore size distribution plot of (g) SNPs, (h) sol-0.06%@77K, and (i) gel-0.25%@77K samples measured using density functional theory (DFT) pore size distribution analysis.

Figure S5: Thermal stability test of (a) SNPs, (b) sol-0.06%@77K aerogel, and (c) gel-0.25%@77K aerogel samples in O_2, CO_2 and N_2 environments using thermogravimetry. The heating rate was 5 °C/min in all cases.

Figure S6: (a) FESEM images of silk-fibroin aerogel prepared using lyophilization of 2, 1, 0.5, and 0.25 wt% aqueous silk solution frozen using (a-d) refrigerator at -80 °C (193 K), and (e-h) liquid nitrogen at -196 °C (77 K).

Figure S7: CO_2 adsorption-desorption isotherms of (a) SNPs, (b) sol-0.06%@77K, and (c) gel-0.25%@77K at various temperatures. Temperature-dependent CO_2 adsorption capacity of (d) SNPs, (e) sol-0.06%@77K, and (f) gel-0.25%@77K as a function of CO_2 pressure.

Figure S8: Magnified CO_2 adsorption-desorption isotherms of SNPs, sol-0.06%@77K and gel-0.25%@77K aerogels at (a) 25 °C and (b) 5 °C in the low partial pressure range (0 to 0.2).

Figure S9: Multi-Cycle CO_2 adsorption performance of various amino acid-based solid sorbents. Figures are adopted with permission from the respective publishers.

Figure S10: Multi-Cycle CO_2 adsorption performance of various amino acid ionic liquids (AAILs) based solid sorbents. Most of the AAILs showed gradually decreasing CO_2 adsorption after several cycles of adsorption and desorption. Figures are adopted with permission from the respective publishers.

Figure S11: Differential adsorption enthalpy (ΔH_{ads}) of (a) SNPs, (b) sol-0.06%@77K, and (c) gel-0.25%@77K as a function of CO_2 adsorption capacity.

Figure S12: Optical image of the gel-0.25%@77K aerogel-loaded U-shaped tube.

Figure S13: XPS survey scan of CO_2-adsrobed SNPs (a) before and (b) after 10 s of monoatomic Ar^+ ion sputtering of energy 200 eV. Fitting of high-resolution C1s spectra (c) before and (d) after 10 s of monoatomic Ar+ ion sputtering of energy 200 eV. The symbols represent the experimental data, and the solid lines represent the fitted data.

Figure S14: ^{13}C solid-state NMR spectra of silk-fibroin aerogel after $^{13}CO_2$ adsorption. Measurement was performed by cross-polarization (with continuous-wave decoupling of ^1H). The magic angle spinning (MAS) rate was (a, b) 10 kHz and (c, d) 15 kHz. During NMR measurement, the sample's temperature inside the rotor was calculated to be ~ 7.5 °C. The $^{13}CO_2$ gas dosing pressure was 1 bar at room temperature, though it is expected to be lower at the measured sample temperature due to pressure drop from additional adsorption and temperature drop. The number of scans was 1024 in each case.

❖ Supplementary discussion:

S1. Synthesis of silk-fibroin nanoparticles (SNPs) from silk cocoon
S2. Characterizations
S3. CO_2 adsorption-desorption study
S4. Discussion on FTIR data

❖ Supplementary Tables:

Table S1: Comparison of the CO_2 adsorption capacity of silk-nano-fibroin-based sorbents with other high-performing amino acid and AAILs-based solid sorbents reported at nearly similar conditions.

Table S2: Comparison of differential adsorption enthalpy (ΔH_{ads}) of silk-nano-fibroin-based sorbents with other state-of-the-art sorbents reported at 1 mmol CO_2/gm sorbent adsorption capacity.

Table S3: Fitting parameter of C1s core-level spectra before and after Ar^+-ion sputtering.

Supplementary Figures

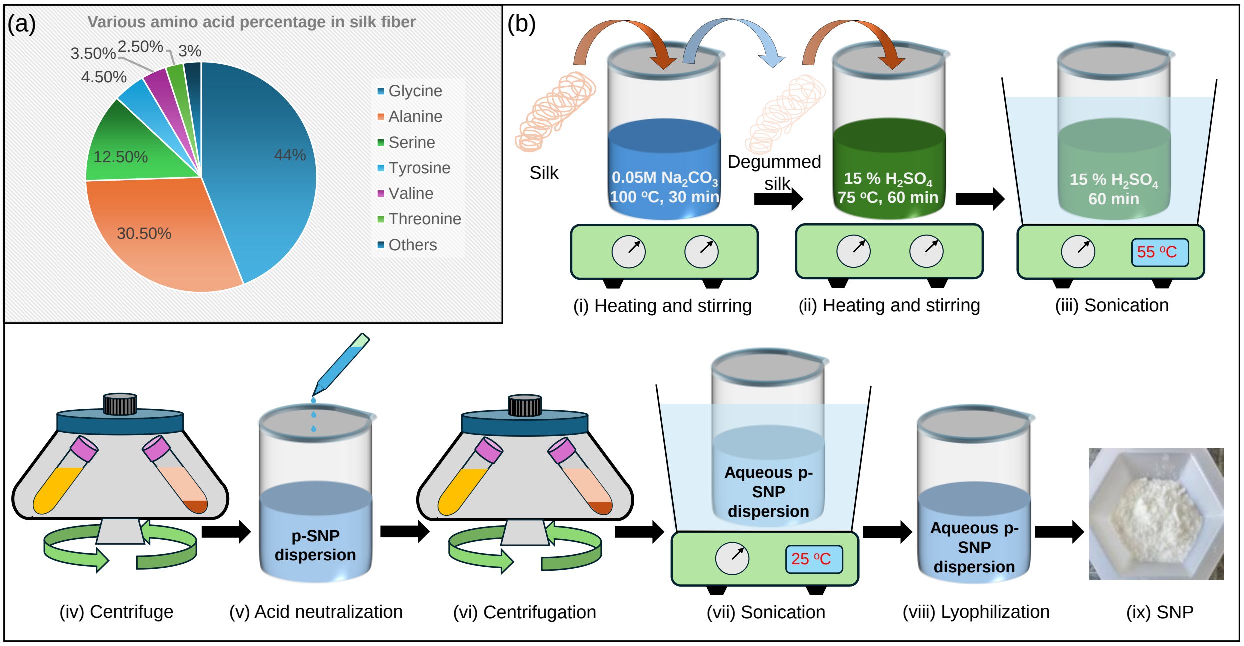

Figure S1: (a) Percentage of various amino acids in the silk-fibroin.[1] (b) A schematic of the porous silk-nanoparticles (SNPs) synthesis from mulberry silk cocoon.

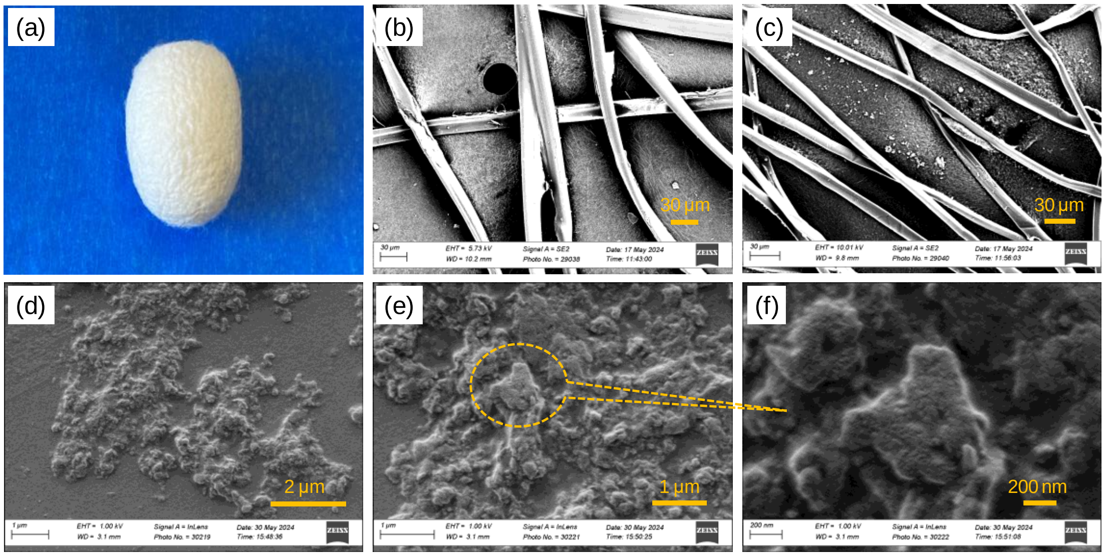

Figure S2: (a) Optical image of the mulberry silk cocoon. (b) Field effect scanning electron microscope (FESEM) image of the raw silk fiber. (c) FESEM image of the degummed silk fiber. (d, e, f) FESEM image of the SNPs at various resolutions.

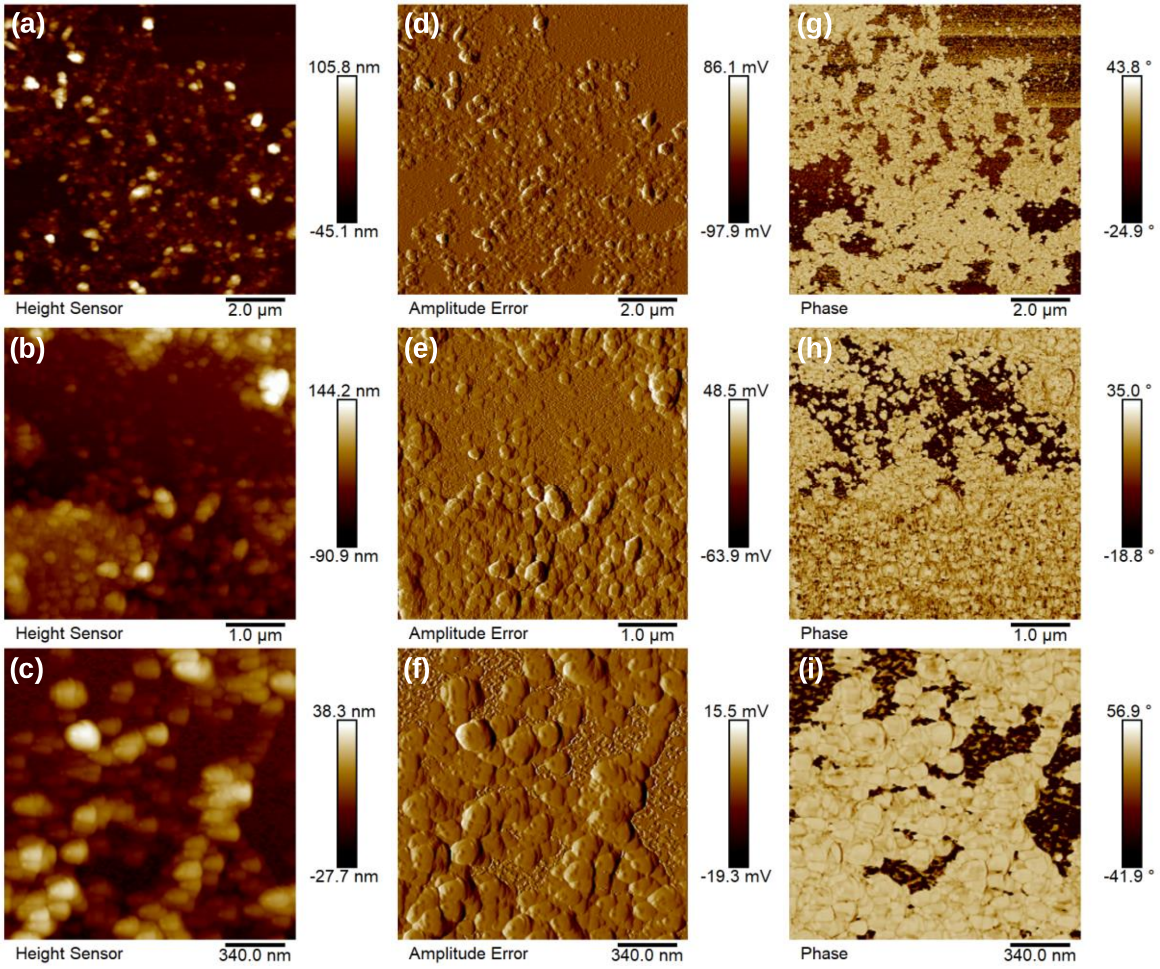

Figure S3: AFM (a, b, c) topography image; simultaneously acquired (d, e, f) amplitude; and (g, h, i) phase image of the SNPs.

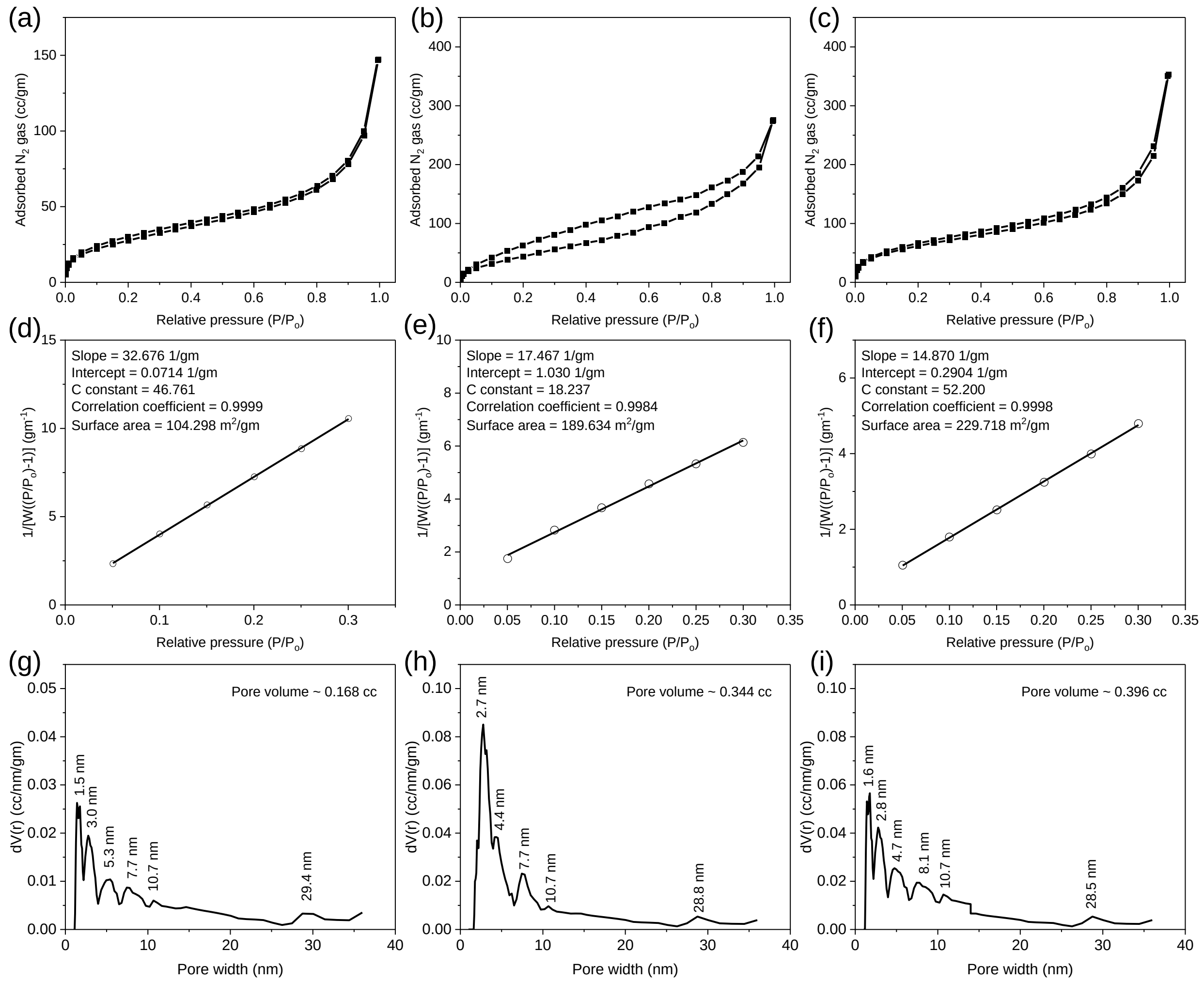

Figure S4: N_2 adsorption-desorption isotherm of (a) SNPs, (b) sol-0.06%@77K, and (c) gel-0.25%@77K aerogels at 77 K. Specific surface area analysis of (d) SNPs, (e) sol-0.06%@77K, and (f) gel-0.25%@77K using the Brunauer–Emmett–Teller (BET) method. Pore size distribution plot of (g) SNPs, (h) sol-0.06%@77K, and (i) gel-0.25%@77K samples measured using density functional theory (DFT) pore size distribution analysis.

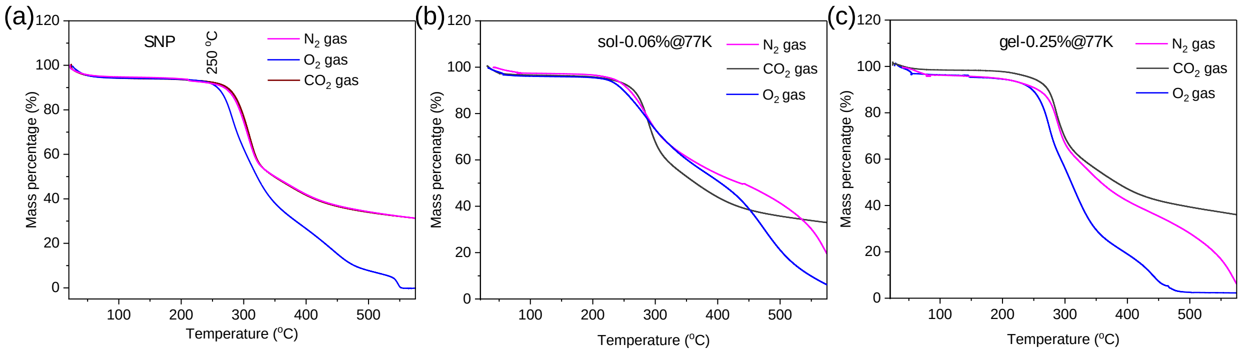

Figure S5: Thermal stability test of (a) SNPs, (b) sol-0.06%@77K aerogel, and (c) gel-0.25%@77K aerogel samples in O_2, CO_2 and N_2 environments using thermogravimetry. The heating rate was 5 °C/min in all cases.

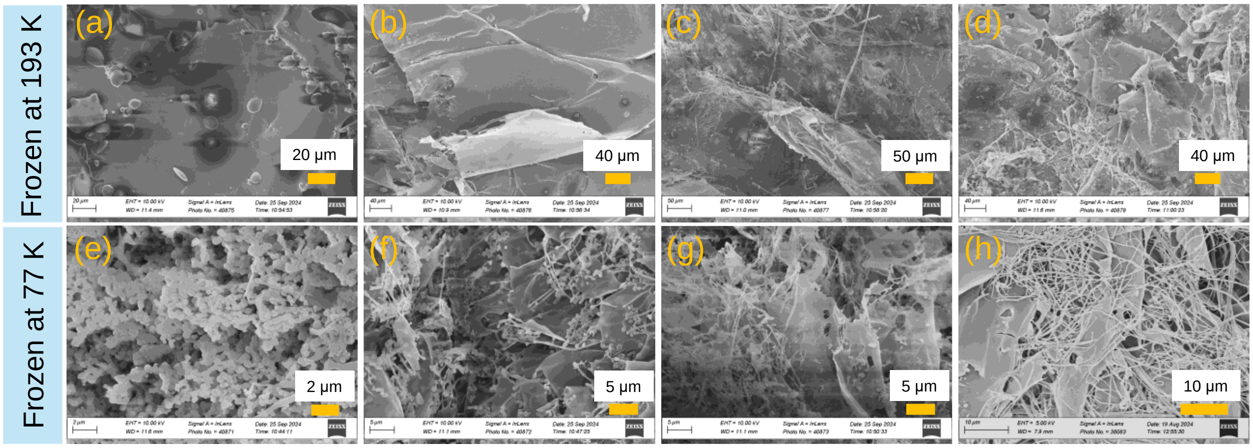

Figure S6: FESEM images of silk-fibroin aerogel prepared using lyophilization of 2, 1, 0.5, and 0.25 wt% aqueous silk solution frozen using (a-d) refrigerator at -80 °C (193 K), and (e-h) liquid nitrogen at -196 °C (77 K).

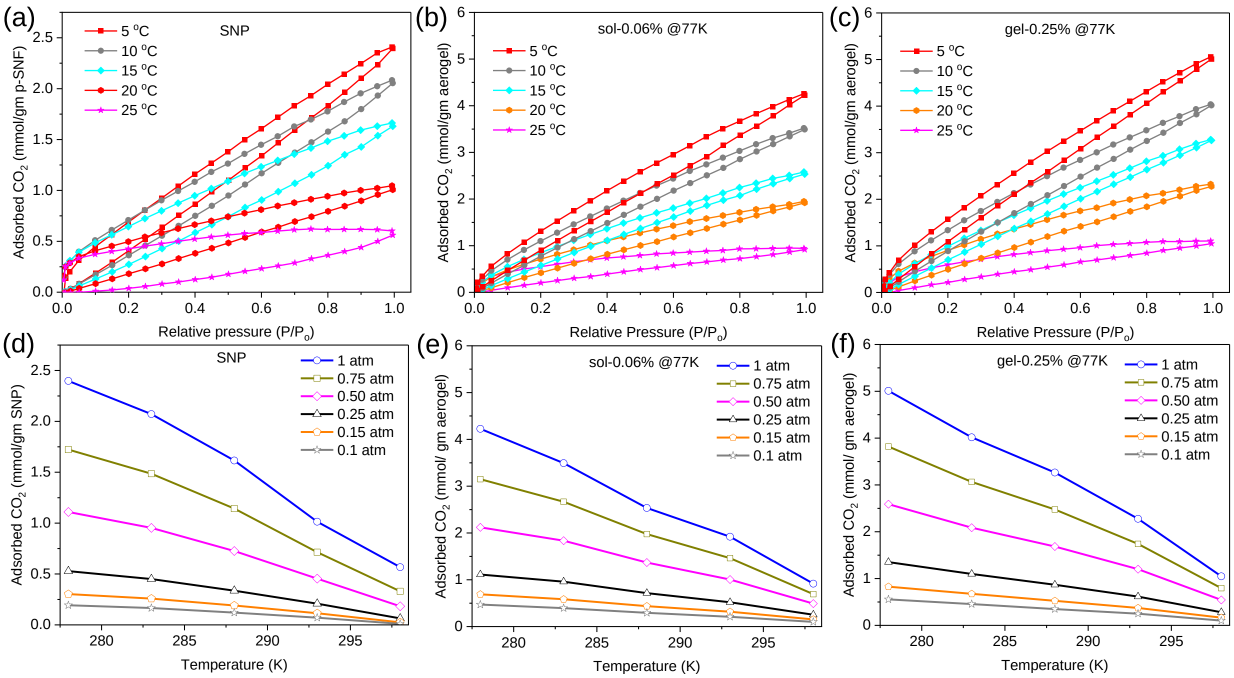

Figure S7: CO_2 adsorption-desorption isotherms of (a) SNPs, (b) sol-0.06%@77K, and (c) gel-0.25%@77K at various temperatures. Temperature-dependent CO_2 adsorption capacity of (d) SNPs, (e) sol-0.06%@77K, and (f) gel-0.25%@77K as a function of CO_2 pressure.

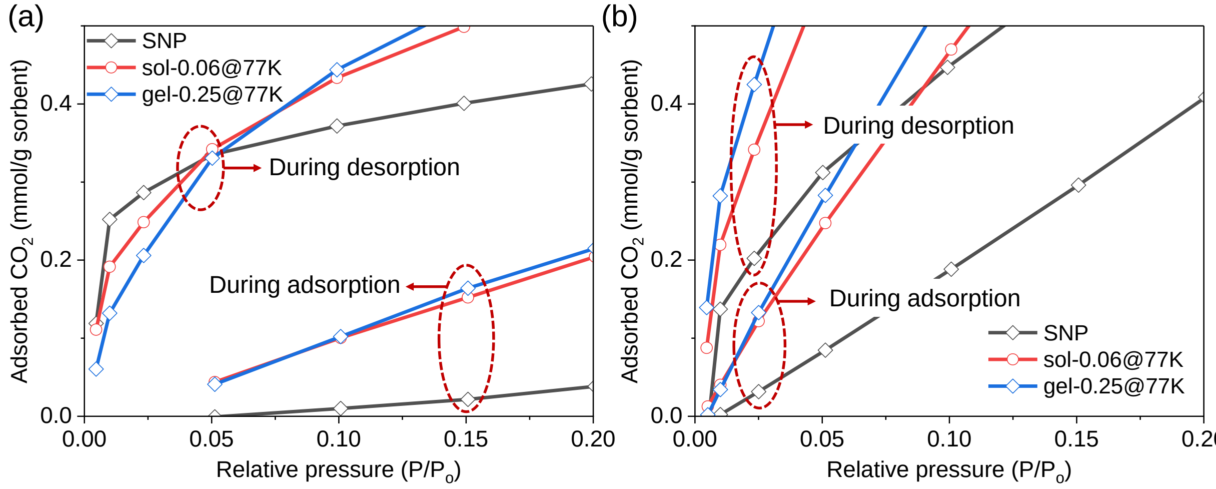

Figure S8: Magnified CO_2 adsorption-desorption isotherms of SNPs, sol-0.06%@77K and gel-0.25%@77K aerogels at (a) 25 °C and (b) 5 °C in the low relative partial pressure range (0 to 0.2).

The enlarged 25 °C isotherm in **Figure S8** showed noticeable scatter in the very low-pressure region because the absolute CO_2 uptake at this temperature is small and approaches the measurement's resolution limit, leading to increased relative noise in the recorded points. Therefore, the measurement at 25 °C was recorded from the relative pressure of 0.05. In contrast, the 5 °C isotherm in **Figure 8(b)** shows higher CO_2 uptake and therefore a stronger signal in the same low-pressure range, which reduces the relative noise and results in a smoother profile.

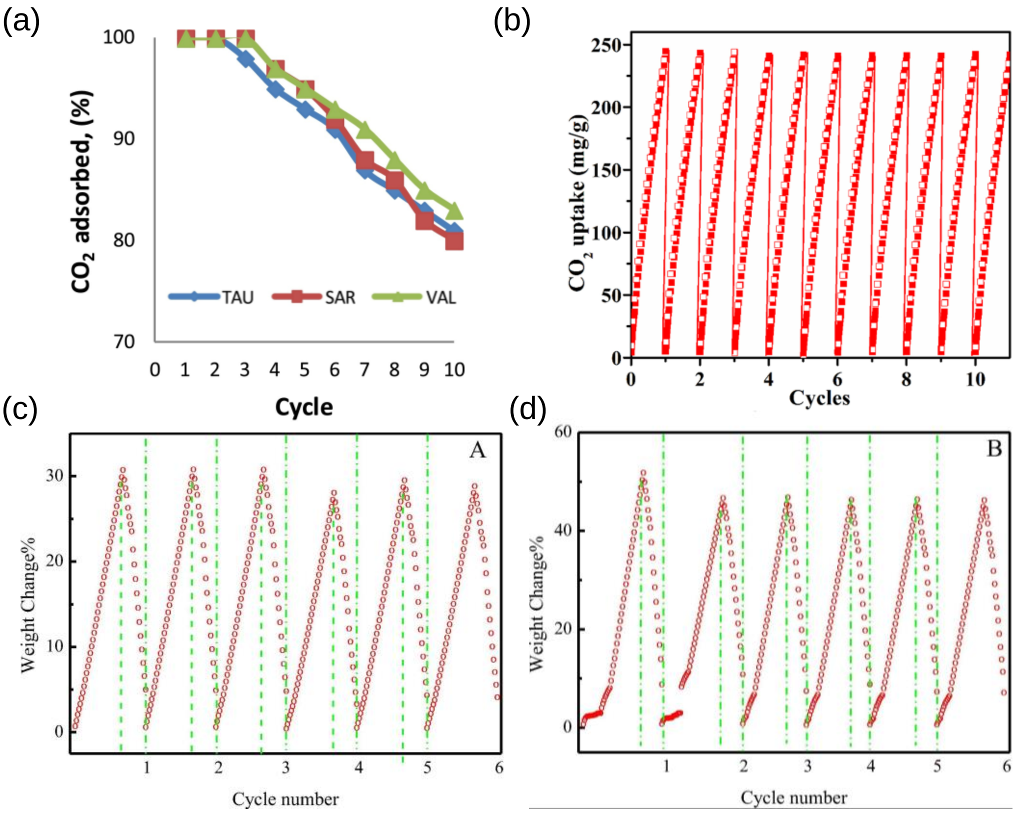

Figure S9: Multi-Cycle CO_2 adsorption performance of various amino acid-based solid sorbents. Figures are adopted with permission from the respective publishers.

(a) Around 20 % drop in adsorption capacity after 10 cycles of adsorption-desorption test using porous taurine, sarcosine and valine. Reproduced with permission.[2] Copyright 2016, Elsevier.

(b) Glycine functionalized covalent triazine framework (BCK-CTF) showed stable CO_2 adsorption capacity in 10 cycles. Reproduced with permission.[3] Copyright 2020, Elsevier.

(c, d) Hyper crosslinked polymer (HCP), HCP(St-DMDAAC), and glycine incorporated HCP(St-DMDAAC) adsorbents showed 2.7% and a 5% drop, respectively, in the CO_2 adsorption capacity after 6 cycles. Reproduced with permission.[4] Copyright 2018, Elsevier.

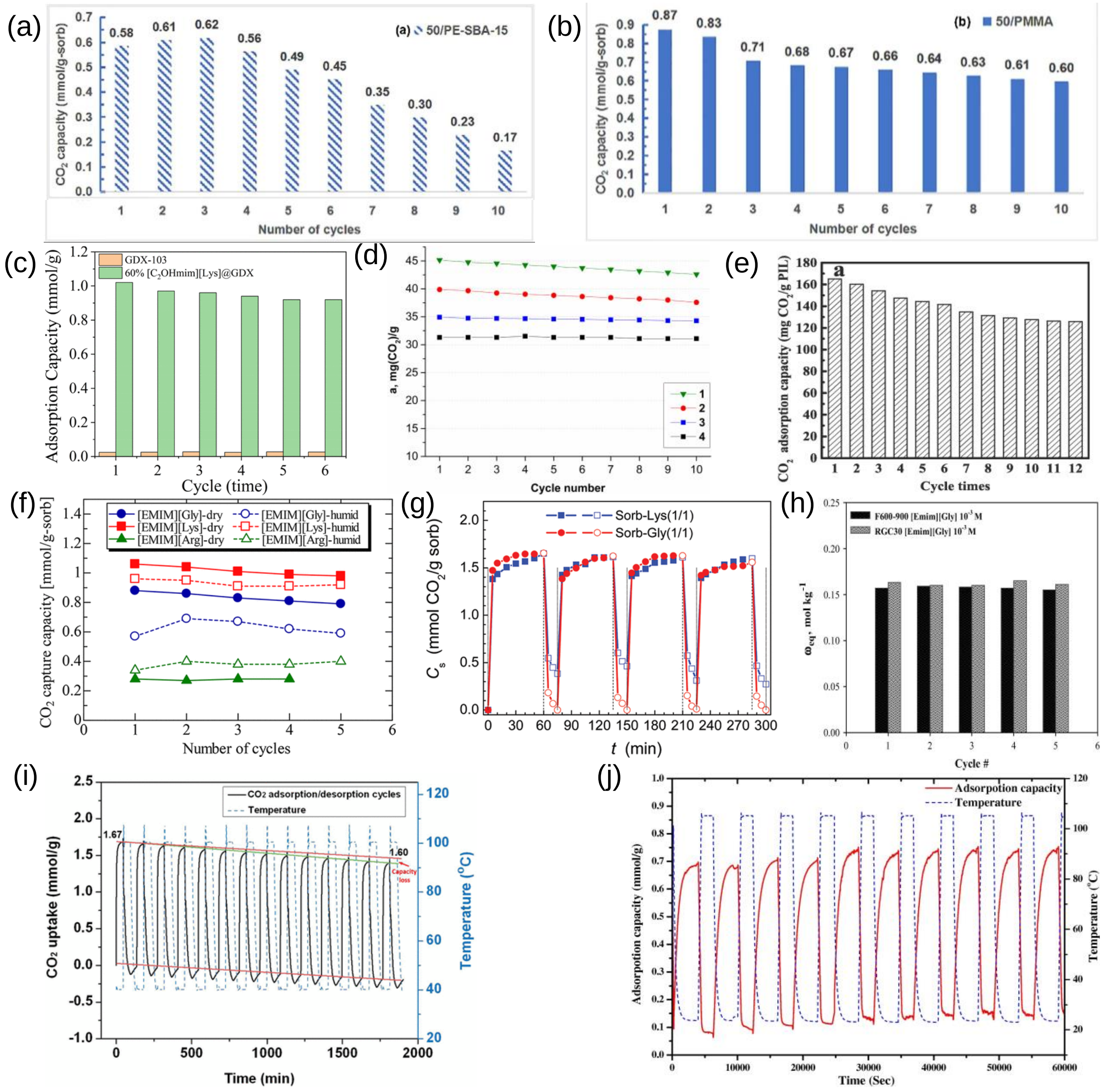

Figure S10: Multi-Cycle CO_2 adsorption performance of various amino acid ionic liquids (AAILs) based solid sorbents. Most of the AAILs showed gradually decreasing CO_2 adsorption after several cycles of adsorption and desorption. Figures are adopted with permission from the respective publishers.

(a, b) Aminoethyl-3-methylimidazolium Lysine, [AEMIM][Lys] functionalized mesoporous silica and poly(methyl methacrylate) (PMMA) shows gradual decrease in CO_2 adsorption capacity during cyclic adsorption desorption study. Reproduced with permission.[5] Copyright 2021, Elsevier.

(c) Amino functionalized ionic liquid [C_2OHmim][Lys] impregnated on a chromatographic column filler poly-divinylbenzene porous spheres, GDX-103 shows gradual decrease in CO_2 adsorption capacity. Reproduced with permission.[6] Copyright 2023, ACS.

(d) 1-ethyl-3-methylimidazolium glycinate [EMIM][Gly] loaded mesoporous silica gel shows gradually decreasing CO_2 adsorption capacity. According to this study high high-temperature regeneration helps in better adsorption capacity, but degrades the sample faster. Reproduced with permission.[7] Copyright 2022, Elsevier.

(e) Porous poly[1-(p-vinylbenzyl)-3-methylimidazolium glycinate], [P([VBMI][Gly])] sorbent shows gradual decrease in CO_2 adsorption capacity due to the destruction of its pore structure during the regeneration process. Reproduced with permission.[8] Copyright 2021, Elsevier.

(f) Glycine and lysine based AAILs functionalized poly(methyl methacrylate) (PMMA) show gradually decreasing CO_2 adsorption capacity in both dry and humid conditions. Arginine based AAIL shows nearly stable CO_2 adsorption in both the conditions. However, [EMIM][Arg] has a very poor adsorption capacity. Reproduced with permission.[9] Copyright 2017, ACS.

(g) N-(3-aminopropyl)aminoethyl tributylphosphonium amino acid, [apaeP_{444}][AA] impregnated mesoporous silica sorbents show gradual decrease in CO_2 adsorption capacity. Reproduced with permission.[10] Copyright 2012, ACS.

(h) [Emim][Gly] AAIL functionalized activated carbon have nearly constant CO_2 adsorption capacity during 4 cycle tests. However, the sorbent has very poor adsorption capacity. Reproduced with permission.[11] Copyright 2015, Elsevier.(i) 4% loss in CO_2 adsorption capacity after 14 cycles for [EMIM][Lys] impregnated PMMA (48.7 wt %) sorbent. [EMIM][Lys] has a thermal degradation temperature of around 200 °C. Reproduced with permission.[12] Copyright 2013, ACS.

(j) Nearly stable CO_2 adsorption capacity observed after 10 cycles of adsorption-desorption test using 1-methyl-3-ethyl-imidazolium lysinate (OMS-IL-Lys) grafted mesoporous silica. However, the sorbent requires a high regeneration temperature of 105 °C due to its high value of differential heat of adsorption (ΔH_{ads} ~ 85.7 kJ/mol). Reproduced with permission.[13] Copyright 2016, Elsevier.

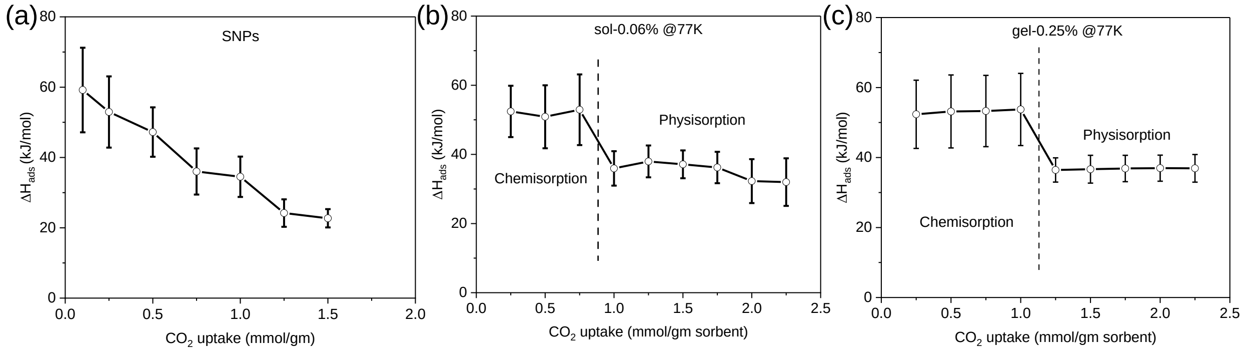

Figure S11: Differential adsorption enthalpy (ΔH_{ads}) of (a) SNPs, (b) sol-0.06%@77K, and (c) gel-0.25%@77K as a function of CO_2 adsorption capacity. The ΔH_{ads} was calculated using the Clausius-Clapeyron approach. The error bars represent the propagated uncertainty from the linear regression fits, derived from the standard error of the slope (SE) and converted to ΔH_{ads} uncertainty using = $R \times SE$, where R is the universal gas constant.

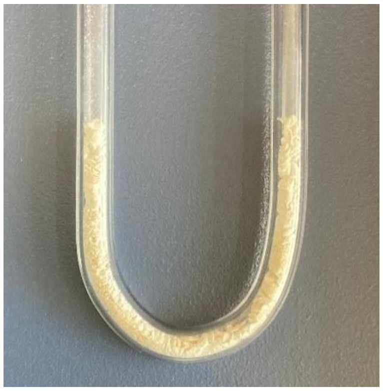

Figure S12: Optical image of the gel-0.25%@77K aerogel-loaded U-shaped tube.

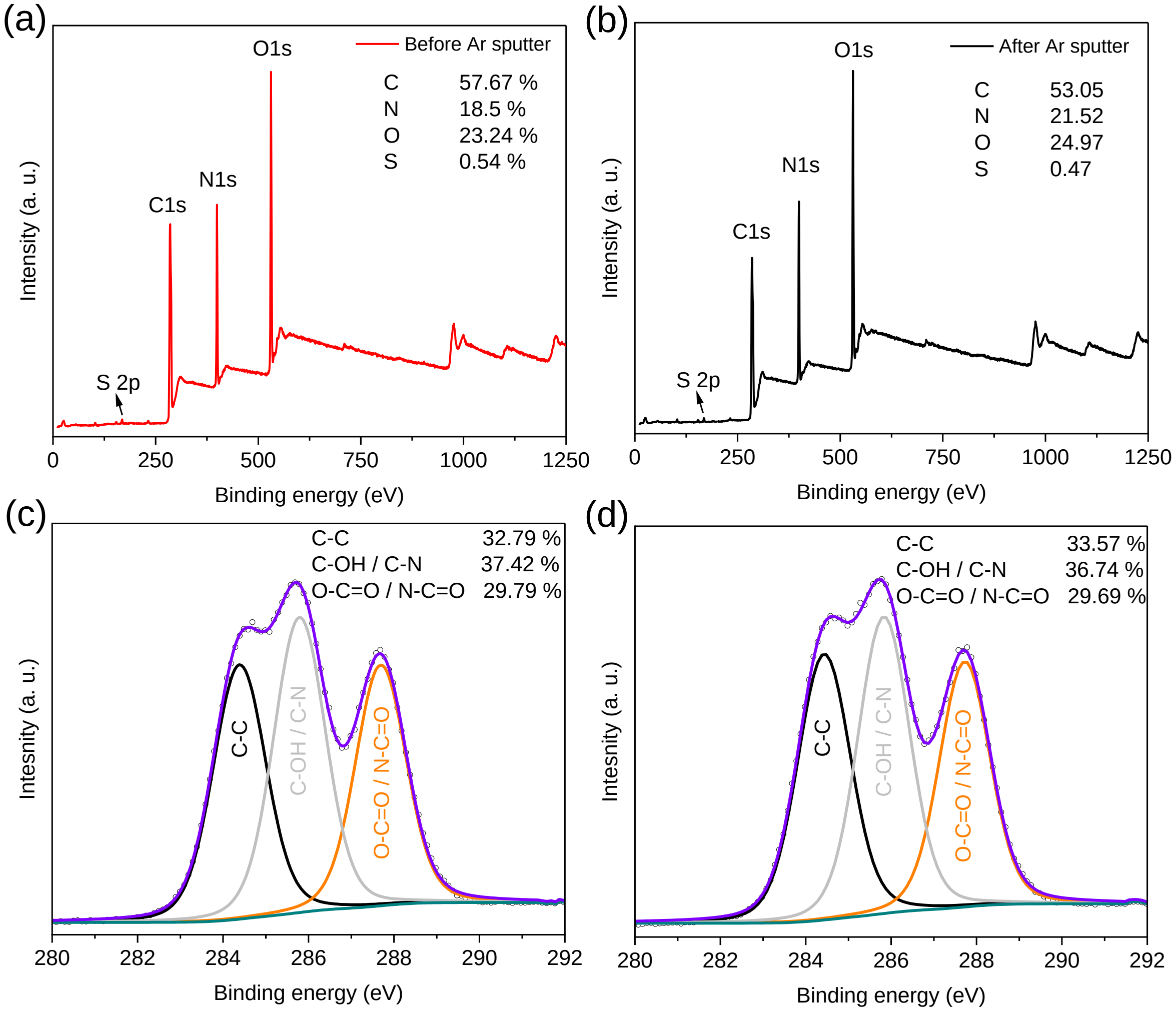

Figure S13: XPS survey scan of CO_2-adsrobed SNPs (a) before and (b) after 10 s of monoatomic Ar^+ ion sputtering of energy 200 eV. Fitting of high resolution C1s spectra (c) before and (d) after 10 s of monoatomic Ar^+ ion sputtering of energy 200 eV. The symbols represent the experimental data, and the solid lines represent the fitted data.

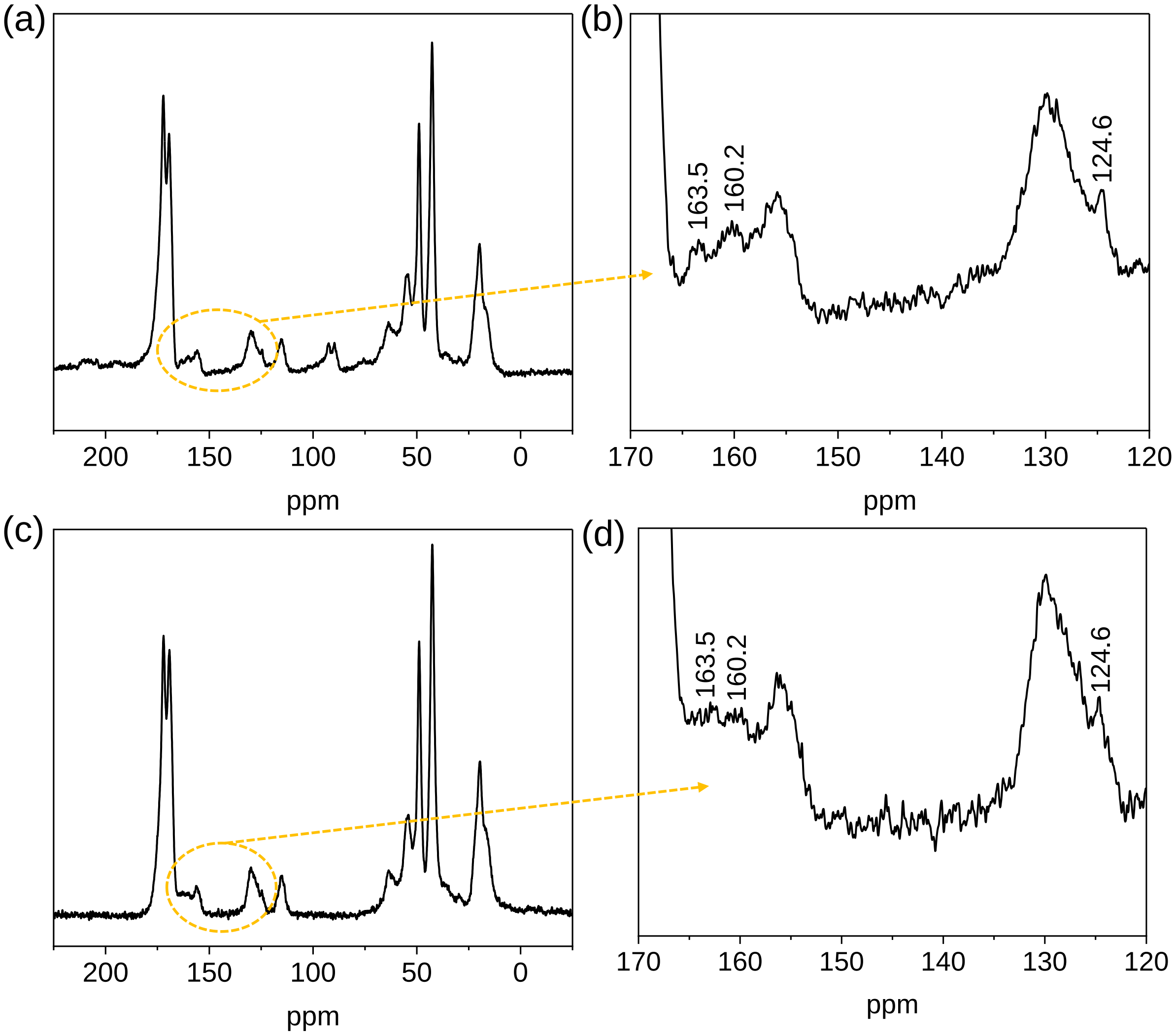

Figure S14: ^{13}C solid-state NMR spectra of silk-fibroin aerogel after $^{13}CO_2$ adsorption. Measurement was performed by cross-polarization (with continuous-wave decoupling of ^{1}H). The magic angle spinning (MAS) rate was (a, b) 10 kHz and (c, d) 15 kHz. During NMR measurement, the sample's temperature inside the rotor was calculated to be 6.9 and 7.5±0.2 °C, respectively. The $^{13}CO_2$ gas dosing pressure was 1 bar at room temperature, though it is expected to be lower at the measured sample temperature due to pressure drop from additional adsorption and temperature drop. The number of scans was 1024 in each case.

Supporting Information File

Table S1: Comparison of the CO_2 adsorption capacity of silk-nano-fibroin-based sorbents with other high-performing amino acid and AAILs-based solid sorbents reported at nearly similar conditions.

AAs/AAILs	Porous solid support	wt % of AA/AAILs w.r.t. sorbent	Effective surface area (m^2/g)	Reported adsorption condition	Adsorption capacity (mmol/g)	References
SNPs	No support	Not applicable	104.27±1.89	1 bar pure CO_2, 25 °C	0.57±0.09	This work
Sol-0.06%@77K	No support	Not applicable	195.37±4.97	1 bar pure CO_2, 25 °C	0.97±0.05	This work
Gel-0.25%@77K	No support	Not applicable	232.31±3.31	1 bar pure CO_2, 25 °C	1.11±0.06	This work
Egg white	Activated Carbon	10	625	1 bar pure CO_2, 25 °C	0.57	Hatta et al.[14]
[APMIM][Lys]	Silica	50	148	1 bar pure CO_2, 30 °C	0.55	Huang et al.[15]
[APMIM][Lys]	PMMA	50	88	1 bar pure CO_2, 30 °C	1.45	Huang et al.[15]
[EMIM][Gly]	UiO-66 (MOF)	5	1102	1 bar pure CO_2, 25 °C	2.5	Xia et al.[16]
[EMIM][Gly]	NU-1000 (MOF)	5	1754	1 bar pure CO_2, 25 °C	1.8	Xia et al.[16]
Arg/PSS	PMMA	25	--	1 bar pure CO_2, 40 °C	1.3	Jiang et al.[17]
Sarcosine	No support	--	--	1 bar pure CO_2, 30 °C	2.63	Chatterjee et al.[2]
Taurine	No support	--	--	1 bar pure CO_2, 30 °C	3.25	Chatterjee et al.[2]
[apaeP_{444}][Lys]	Silica	50	150	1 bar pure CO_2, 25 °C	1.87	Ren et al.[10]

[apaeP_{444}][Ala]	Silica	50	--	1 bar pure CO_2, 25 °C	1.46	Ren et al.[10]
[apaeP_{444}][Gly]	Silica	50	137	1 bar pure CO_2, 25 °C	1.46	Ren et al.[10]
[apaeP_{444}][His]	Silica	50	--	1 bar pure CO_2, 25 °C	1.46	Ren et al.[10]
[apaeP_{444}][Asp]	Silica	50	--	1 bar pure CO_2, 25 °C	1.46	Ren et al.[10]
[EMIM][Gly]	PMMA	50	--	1 bar pure CO_2, 40 °C	1.53	Wang et al.[12]
[EMIM][Ala]	PMMA	50	--	1 bar pure CO_2, 40 °C	1.38	Wang et al.[12]
[EMIM][Arg]	PMMA	50	--	1 bar pure CO_2, 40 °C	1.01	Wang et al.[12]
[EMIM][Lys]	PMMA	50	27	1 bar pure CO_2, 40 °C	1.67	Wang et al.[12]
Gly	BCK-CTF	--	1720	1 bar pure CO_2, 25 °C	2.61	Dong et al. [18]

1-aminopropyl-3-methylimidazolium lysine ([APMIM][Lys]), polystyrene sulfonate (PSS), tetramethylammonium glycinate ([N_{1111}][Gly]), *N*-(3-aminopropyl)aminoethyl tributylphosphonium amino acid salt ([apaeP_{444}][AA]), covalent triazine frameworks (CTFs), bis(4-cyanophenyl)ketone (BCK).

Table S2: Comparison of differential adsorption enthalpy (ΔH_{ads}) of silk-nano-fibroin-based sorbents with other state-of-the-art sorbents reported at 1 mmol CO_2/gm sorbent adsorption capacity.

Sorbents	Differential adsorption enthalpy (ΔH_{ads}) (-kJ/mol)	References
SNPs	53.12±5.74	This work
Sol-0.06%@77K	52.08±5.19	This work
Gel-0.25%@77K	53.13±5.08	This work
Carbonaceous samples	23	Fan et al.[19]
Zeolite	58	Bae et al.[20]
SBA-15 (porous silica)	20	Mohamedali et al.[21]
PP1-2 (Porous polymer)	20	Xu et al.[22]

Amine@PP1-2-tren (Amine modified porous polymer)	45	Xu et al.[22]
PMMA	44	Huang et al.[15]
MNNsCya-DETA (polyamine-appended, cyanuric acid–stabilized melamine)	53	Mao et al.[23]
Amine@MOF (tetraamine-appended metal–organic frameworks)	99	Kim et al.[24]
MOF-177 (metal–organic frameworks)	13	Philip et al.[25]
[Emim][Gly]@porous silica	87.7	Sheshkovas et al.[7]
Gly@BCK-CTF (glycine-functionalized covalent triazine framework)	33.3	Dong et al.[3]
[Emim][Ala]@MOF-177	14	Philip et al.[25]
[Emim][Gly]@MOF-177	16	Philip et al.[25]
[APMIM][Lys]@PE-SBA-15	25	Huang et al.[15]

Table S3: Fitting parameter of C1s core-level spectra before and after Ar^+-ion sputtering.

Name	Before Ar sputter			After Ar sputter		
	Position (eV)	FWHM	Area %	Position (eV)	FWHM	Area %
C-C	284.39	1.46	32.79	284.43	1.46	33.57
C-OH / C-N	285.79	1.43	37.42	285.83	1.43	36.74
O-C=O / N-C=O	287.69	1.40	29.79	287.72	1.40	29.69

S1. Synthesis of silk-fibroin nanoparticles (SNPs) from silk cocoon

SNPs was prepared using the partial acid hydrolysis method, **Figure S1(b)**.[26] At first, mulberry silk cocoons were cut into small pieces and boiled in a 0.05 M Na_2CO_3 aqueous solution for 30 min (i). After removing the outer sericin layer by boiling, the silk was washed with cold water several times. The boiling and washing process was repeated one more time, and the degummed silk was dried in air. In the hydrolysis method, 0.25 gm of degummed silk was taken in a 100 ml glass beaker and 40 ml of 5 weight % H_2SO_4 solution was added into it. Then the beaker was placed on a hot plate and heated to 75 °C. During the acid hydrolysis process the surrounding temperature and humidity were 20-22 °C and 35-40 %, respectively. (ii). The silk dispersion was continuously stirred using a magnetic stirrer while heating. The H_2SO_4 weight % in the solution was increased to 15 % (7.5, 10, 12.5 and 15 %) in 4 steps by adding the equal amount of 98 weight % H_2SO_4 at 5 minutes interval. The solution was stirred on the hot plate for an additional 40 min. Then, the solution was placed on a sonicator bath preheated to 55 °C and sonicated for 1 hour (iii). After that, the dispersed silk was separated from the acid by centrifugation at 10000 rpm for 3 min (iv). The separated silk was taken in a glass beaker with 10 ml of water and neutralized using 0.5 M NaOH solution (v). Then the silk-fibroin was washed 3 times with DI water using centrifugation at 10000 rpm for 3 min to remove the salt generated from the acid neutralization (vi). The cleaned silk was then dispersed in water again and placed in a sonicator bath for 30 min at room

temperature (vii). After sonication, the dispersion was frozen at -80 °C. Finally, the frozen dispersion was lyophilized at -48 °C to obtain the SNPs (viii-ix).

S2. Characterizations

The room temperature X-ray diffraction (XRD) patterns of the degummed silk, SNPs, sol-0.06%@77K and gel-0.25%@77K were studied using a Cu-Kα X-ray diffractometer (Bruker D8 Discovery). Field-emission scanning electron microscopy (FESEM) images were collected using a scanning electron microscope (SEM, Model: Zeiss 1530). Atomic force microscopy (AFM) measurement was performed using a Bruker Dimension Icon AFM. Thermo Al-Kα X-ray photoelectron spectrometer was used for the X-ray photoemission spectroscopy (XPS) study of the SNPs. For the XPS and AFM studies, the SNPs was dispersed in water and drop-casted on microscopic glass slides. After drop casting, the glass slides were heated in air at 90 °C for 15 min. The particle size measurement was performed using the aqueous SNPs dispersion by a Zetasizer (Malvern Nano ZSP). The thermogravimetry analysis (TGA) of the SNPs, sol-0.06%@77K and gel-0.25%@77K was studied from room temperature to 575 °C in O_2, N_2 and CO_2 gas environments using a thermogravimeter (Model TGA Q550). Nitrogen adsorption desorption measurements were carried out at 77 K using a Quantachrome (Model: autosorb iQ7) instrument. The average specific surface area of the silk-fibroin-based sorbents was measured by the Brunauer–Emmett–Teller (BET) method, with n = 3 independent measurements on the same sample. Room temperature Fourier Transform Infrared Spectroscopy (FTIR) was studied in ambient atmosphere using an FTIR spectrometer (Thermo Fisher Scientific: Nicolet iS10). To study the FTIR spectra of SNPs before CO_2 adsorption, it was heated in an oven at 95 °C for 30 min to desorb chemisorbed CO_2 that occurred during air exposure, and were instantly dispersed in hot water (95 °C) to avoid CO_2 adsorption before FTIR measurement. To study the FTIR spectra of SNPs after CO_2 adsorption, the CO_2-exposed sample was dispersed in water at room temperature, and its FTIR spectrum was recorded. The obtained spectrum was similar to that of the air-exposed sample measured in air.

Raman spectra were collected using a Horiba LabRAM HR Evolution confocal Raman microscope. A 100 mW 532 nm laser was used as an excitation light source along with an 1800 line/mm grating spectrometer. The laser power was adjusted to 1% by a filter. A 50× long-working-distance objective with an N.A. of 0.5 was used for all measurements. All spectrum was collected under the following conditions: an acquisition time of 15 s, three accumulations, and a spectral range of 850–1200 cm^{-1}. The raw spectra were processed using a Python-based routine. First, baseline correction was performed using an Asymmetric Least Squares (ALS) smoothing algorithm with a smoothing parameter (λ) of 1×10^4, an asymmetry parameter (p) of 0.01, and 10 iterations to estimate and subtract the baseline. Subsequently, the baseline-corrected spectra were normalized and further smoothed using a Savitzky–Golay filter (window length = 11, polynomial order = 5) to reduce noise while preserving peak features.

Solid-state nuclear magnetic resonance (NMR) spectroscopy measurements were performed using the silk-fibroin aerogel before and after CO_2 gas adsorption. To enhance the signal-to-noise ratio and selectively track the carbon atom of the adsorbed CO_2 gas against the significantly larger

amount of background carbon from the silk-fibroin amino acids, we used $^{13}CO_2$ gas (99.0 atom% ^{13}C, Sigma Aldrich) at atmospheric pressure. To study the ^{13}C spectra before adsorption, the as-synthesized silk-fibroin aerogel was loaded into a Bruker 4 mm rotor, and both the sample-loaded rotor and cap (not sealed) were placed in a gas sampling bag. The gas sampling bag was partially filled with N_2 gas and was placed inside an oven at 90 °C for 15 minutes to release any surface-adsorbed gas or moisture. The N_2 gas inside the plastic bag was replaced once, and the bag was heated in the oven for 15 minutes. Then, the sample inside the bag was cooled to room temperature, and the rotor was sealed in an N_2 environment at atmospheric pressure using the macor cap inside the bag (with hand pressure applied from outside), without exposure to air. The sealed rotor was stored inside a gas sampling bag in the N_2 environment until the measurement was taken. After studying the ^{13}C spectra before CO_2 adsorption, the sample-loaded rotor and cap (separated) were placed in a gas sampling bag and heated in N_2 gas at 90 °C for 15 minutes. Then, the gas sampling bag was connected to a vacuum pump for 5 minutes. Finally, a small amount of $^{13}CO_2$ gas was purged into the gas sampling bag to expose the sample to $^{13}CO_2$. The rotor cap was then sealed within the bag at room temperature and atmospheric pressure, without exposure to air.

All NMR measurements were performed at 11.74 T (^1H frequency = 500.22 MHz and ^{13}C frequency = 125.79 MHz) using a Bruker 4 mm probe with a magic angle spinning (MAS) rate of 10 or 15 kHz, as specified in the figure caption. Cross-polarized magic-angle spinning (CP/MAS) ^{13}C NMR spectra were acquired with SPINAL-64 proton decoupling (~94 kHz RF field, 2.5 µs ^1H and 2.65 µs ^{13}C 90° pulses) and a contact time of 2000 µs, averaging 1024 scans. ^1H and ^{13}C chemical shifts were referenced secondarily to ^1H-adamantane at 1.85 ppm, as reported previously.[27] For a fixed MAS rate and probe temperature, the temperature of the sample inside the rotor was measured using KBr-adamantane before the measurement. The sample was cooled by cooling the bearing gas, and at each temperature, it was equilibrated for 15 minutes before starting the measurement. A set probe temperature of -3.1±0.2 °C at a MAS rate of 10 kHz resulted in a sample temperature of 6.9±0.2 °C. For the same sample temperature ~7.5±0.2 °C at a 15 kHz MAS rate, the probe temperature was set to -15±0.2 °C.

S3. CO_2 adsorption-desorption study

Equilibrium CO_2 Adsorption–Desorption Isotherms (Volumetric Method):

To study the CO_2 adsorption capacity, the sorbents were heated in vacuum at 100 °C for 1 h to remove any adsorbed CO_2 gas, moisture, or any other removable surface components. CO_2 adsorption-desorption isotherms of silk-fibroin-based sorbents were studied using a Quantachrome (Model: autosorb iQ7) instrument. The equilibrium CO_2 uptake (mmol/g) obtained from the volumetric analysis was calculated by converting the absorbed gas volume (V, mL at STP) to millimoles using the relation n = V/22,400, where 22,400 mL corresponds to the volume of 1 mol of gas at STP. The temperature of the sample cell was controlled using a liquid bath temperature controller (Model JULABO 200F). The multi-cycle stability of the aerogels was evaluated by repeating the CO_2 adsorption-desorption isotherms at 5 °C using the same Quantachrome (Model: autosorb iQ7) instrument. To study the multi-cycle moisture stability, the aerogel gel-0.25%@77K was exposed overnight to air at 60 to 80% relative humidity at around 22-23 °C; the next day, it was outgassed by vacuum heating at 100 °C for 30 min before CO_2 adsorption-desorption isotherm

measurements. The average CO_2 adsorption capacity values reported at 1 atm and 25 °C correspond to the equilibrium loading determined from the volumetric adsorption isotherm, with n = 3 independent measurements on the same sample.

CO_2 Adsorption–Desorption Kinetics in Flowing Mixed Gas:

To study the adsorption kinetics and understand the adsorption capacity in humid gas, around 70 mg of the gel-0.25%@77K aerogel was loaded in a U-shaped quartz tube (**Figure S12**, ID ~ 3 mm, OD ~ 5 mm), and ~13.3 % CO_2 balanced N_2 gas was passed through the U-tube at a total flow rate of 8.3 SCCM, and the output gas from the adsorption-desorption tube was analyzed using gas chromatography (GC, Inficon Micro GC Fusion). For the study, first, the U-shaped tube was dipped into a 60 °C water bath for 10 minutes to desorb any adsorbed CO_2 gas. After that, the U-shaped tube was transferred to a water bath at 5 °C and kept for ~10 minutes. After the CO_2 adsorption step, the tube was transferred to the 60 °C water bath again for regeneration. During this adsorption-desorption cycle, the gas concentration at the outlet of the sample tube was monitored using the GC continuously. To study the CO_2 adsorption kinetics in the presence of humidity, the dry gas mixture was first passed through two conical flasks containing water to gain moisture. The relative humidity in the gas stream was measured using a humidity meter. The relative humidity in the gas stream at 5 °C was 83±2%. The humid gas was passed through the sample tube, and the adsorption kinetics were studied. To study desorption kinetics, after adsorption, the gas in the tube was switched back to dry gas, and the U-shaped tube was transferred to a hot water bath at 60 °C for sorbent regeneration.

CO_2 Desorption Kinetics Study using TGA:

To study CO_2 desorption kinetics in a CO_2 gas environment, first, the CO_2 adsorption was performed by keeping the gel-0.25%@77K aerogel in a 1 atm CO_2 environment at 23 °C for 15 minutes. Then, its mass change was measured during regeneration in a 1 atm CO_2 environment at 60 °C using TGA (Model TGA Q550).

S4. FTIR analysis

The FTIR spectra, **Figure 3(c)**, show the absorption peaks at 3281, 3074, 2930, 1617, 1511, 1439, 1261, 1230, 1163, 1063, 976, and 693 cm^{-1}. The peaks at 3281 and 3074 cm^{-1} are associated with the N−H symmetric and antisymmetric stretching mode vibration of the amine groups.[6,28] The peak 2930 cm^{-1} is mainly associated with the aliphatic C–H stretching.[29] The broad and intense peak at 1617 cm^{-1} is due to the bending vibration of N–H bond overlapped with the carbonyl bond C=O.[13] The peak at 1511 cm^{-1} represents the combination of the C-N stretching mode vibration and N-H bending mode vibration in amide II.[30] The peak at 1439 is associated with the bending vibration of CH_3 in glycine and alanine.[4] The peak at 1165 cm^{-1} is caused by the C–N stretching in tyrosine.[31] The C–N stretching vibration of glycine appears at 1063 cm^{-1} which also overlaps with to the C–O bond stretching vibration mode of -OH group.[3] The peak at 693 cm-1 is associated with the at COO^- bending.[32] Overall, the FTIR spectra show the presence of amino and carboxyl groups and suggest the presence of amino acids including glycine and alanine in the silk-fibroin-based sorbent.

References:

(1) Babu, K. M. 11 - Silk Fibres – Structure, Properties and Applications. In *Handbook of Natural Fibres (Second Edition)*; Kozłowski, R. M., Mackiewicz-Talarczyk, M., Eds.; Woodhead Publishing Series in Textiles; Woodhead Publishing, 2020; pp 385–416. https://doi.org/10.1016/B978-0-12-818398-4.00013-X.

(2) Chatterjee, S.; Rayalu, S.; Kolev, S. D.; Krupadam, R. J. Adsorption of Carbon Dioxide on Naturally Occurring Solid Amino Acids. *Journal of Environmental Chemical Engineering* **2016**, *4* (3), 3170–3176. https://doi.org/10.1016/j.jece.2016.06.007.

(3) Dong, B.; Wang, D.-Y.; Wang, W.-J.; Tian, X.-L.; Ren, G. Post Synthesis of a Glycine-Functionalized Covalent Triazine Framework with Excellent CO_2 Capture Performance. *Microporous and Mesoporous Materials* **2020**, *306*, 110475. https://doi.org/10.1016/j.micromeso.2020.110475.

(4) Ouyang, H.; Guo, L.; Li, C.; Chen, X.; Jiang, B. Fabrication and Adsorption Performance for CO_2 Capture of Advanced Nanoporous Microspheres Enriched with Amino Acids. *Journal of Colloid and Interface Science* **2018**, *532*, 433–440. https://doi.org/10.1016/j.jcis.2018.07.121.

(5) Huang, Z.; Karami, D.; Mahinpey, N. Study on the Efficiency of Multiple Amino Groups in Ionic Liquids on Their Sorbents Performance for Low-Temperature CO_2 Capture. *Chemical Engineering Research and Design* **2021**, *167*, 198–206. https://doi.org/10.1016/j.cherd.2021.01.016.

(6) Wu, J.; Yang, Z.; Xie, J.; Zhu, P.; Wei, J.; Jin, R.; Yang, H. Porous Polymer Supported Amino Functionalized Ionic Liquid for Effective CO_2 Capture. *Langmuir* **2023**, *39* (7), 2729–2738. https://doi.org/10.1021/acs.langmuir.2c03217.

(7) Sheshkovas, A. Z.; Veselovskaya, J. V.; Rogov, V. A.; Kozlov, D. V. Thermochemical Study of CO_2 Capture by Mesoporous Silica Gel Loaded with the Amino Acid Ionic Liquid 1-Ethyl-3-Methylimidazolium Glycinate. *Microporous and Mesoporous Materials* **2022**, *341*, 112113. https://doi.org/10.1016/j.micromeso.2022.112113.

(8) Sun, L.; Gao, M.; Tang, S. Porous Amino Acid-Functionalized Poly(Ionic Liquid) Foamed with Supercritical CO_2 and Its Application in CO_2 Adsorption. *Chemical Engineering Journal* **2021**, *412*, 128764. https://doi.org/10.1016/j.cej.2021.128764.

(9) Uehara, Y.; Karami, D.; Mahinpey, N. Effect of Water Vapor on CO_2 Sorption–Desorption Behaviors of Supported Amino Acid Ionic Liquid Sorbents on Porous Microspheres. *Ind. Eng. Chem. Res.* **2017**, *56* (48), 14316–14323. https://doi.org/10.1021/acs.iecr.7b02771.

(10) Ren, J.; Wu, L.; Li, B.-G. Preparation and CO_2 Sorption/Desorption of N-(3-Aminopropyl)Aminoethyl Tributylphosphonium Amino Acid Salt Ionic Liquids Supported into Porous Silica Particles. *Ind. Eng. Chem. Res.* **2012**, *51* (23), 7901–7909. https://doi.org/10.1021/ie2028415.

(11) Erto, A.; Silvestre-Albero, A.; Silvestre-Albero, J.; Rodríguez-Reinoso, F.; Balsamo, M.; Lancia, A.; Montagnaro, F. Carbon-Supported Ionic Liquids as Innovative Adsorbents for CO_2 Separation from Synthetic Flue-Gas. *Journal of Colloid and Interface Science* **2015**, *448*, 41–50. https://doi.org/10.1016/j.jcis.2015.01.089.

(12) Wang, X.; Akhmedov, N. G.; Duan, Y.; Luebke, D.; Hopkinson, D.; Li, B. Amino Acid-Functionalized Ionic Liquid Solid Sorbents for Post-Combustion Carbon Capture. *ACS Appl. Mater. Interfaces* **2013**, *5* (17), 8670–8677. https://doi.org/10.1021/am402306s.

(13) Hiremath, V.; Jadhav, A. H.; Lee, H.; Kwon, S.; Seo, J. G. Highly Reversible CO_2 Capture Using Amino Acid Functionalized Ionic Liquids Immobilized on Mesoporous Silica. *Chemical Engineering Journal* **2016**, *287*, 602–617. https://doi.org/10.1016/j.cej.2015.11.075.
(14) Mohamed Hatta, N. S.; Hussin, F.; Gew, L. T.; Aroua, M. K. Enhancing Surface Functionalization of Activated Carbon Using Amino Acids from Natural Source for CO_2 Capture. *Separation and Purification Technology* **2023**, *313*, 123468. https://doi.org/10.1016/j.seppur.2023.123468.
(15) Huang, Z.; Mohamedali, M.; Karami, D.; Mahinpey, N. Evaluation of Supported Multi-Functionalized Amino Acid Ionic Liquid-Based Sorbents for Low Temperature CO_2 Capture. *Fuel* **2022**, *310*, 122284. https://doi.org/10.1016/j.fuel.2021.122284.
(16) Xia, X.; Hu, G.; Li, W.; Li, S. Understanding Reduced CO_2 Uptake of Ionic Liquid/Metal–Organic Framework (IL/MOF) Composites. *ACS Appl. Nano Mater.* **2019**, *2* (9), 6022–6029. https://doi.org/10.1021/acsanm.9b01538.
(17) Jiang, B.; Wang, X.; Gray, M. L.; Duan, Y.; Luebke, D.; Li, B. Development of Amino Acid and Amino Acid-Complex Based Solid Sorbents for CO_2 Capture. *Applied Energy* **2013**, *109*, 112–118. https://doi.org/10.1016/j.apenergy.2013.03.070.
(18) Dong, B.; Wang, D.-Y.; Wang, W.-J.; Tian, X.-L.; Ren, G. Post Synthesis of a Glycine-Functionalized Covalent Triazine Framework with Excellent CO_2 Capture Performance. *Microporous and Mesoporous Materials* **2020**, *306*, 110475. https://doi.org/10.1016/j.micromeso.2020.110475.
(19) Fan, X.; Zhang, L.; Zhang, G.; Shu, Z.; Shi, J. Chitosan Derived Nitrogen-Doped Microporous Carbons for High Performance CO_2 Capture. *Carbon* **2013**, *61*, 423–430. https://doi.org/10.1016/j.carbon.2013.05.026.
(20) Bae, T.-H.; Hudson, M. R.; Mason, J. A.; Queen, W. L.; Dutton, J. J.; Sumida, K.; Micklash, K. J.; Kaye, S. S.; Brown, C. M.; Long, J. R. Evaluation of Cation-Exchanged Zeolite Adsorbents for Post-Combustion Carbon Dioxide Capture. *Energy Environ. Sci.* **2012**, *6* (1), 128–138. https://doi.org/10.1039/C2EE23337A.
(21) Mohamedali, M.; Ibrahim, H.; Henni, A. Imidazolium Based Ionic Liquids Confined into Mesoporous Silica MCM-41 and SBA-15 for Carbon Dioxide Capture. *Microporous and Mesoporous Materials* **2020**, *294*, 109916. https://doi.org/10.1016/j.micromeso.2019.109916.
(22) Xu, C.; Bacsik, Z.; Hedin, N. Adsorption of CO_2 on a Micro-/Mesoporous Polyimine Modified with Tris(2-Aminoethyl)Amine. *J. Mater. Chem. A* **2015**, *3* (31), 16229–16234. https://doi.org/10.1039/C5TA01321F.
(23) Mao, H.; Tang, J.; Day, G. S.; Peng, Y.; Wang, H.; Xiao, X.; Yang, Y.; Jiang, Y.; Chen, S.; Halat, D. M.; Lund, A.; Lv, X.; Zhang, W.; Yang, C.; Lin, Z.; Zhou, H.-C.; Pines, A.; Cui, Y.; Reimer, J. A. A Scalable Solid-State Nanoporous Network with Atomic-Level Interaction Design for Carbon Dioxide Capture. *Science Advances* **2022**, *8* (31), eabo6849. https://doi.org/10.1126/sciadv.abo6849.
(24) Kim, E. J.; Siegelman, R. L.; Jiang, H. Z. H.; Forse, A. C.; Lee, J.-H.; Martell, J. D.; Milner, P. J.; Falkowski, J. M.; Neaton, J. B.; Reimer, J. A.; Weston, S. C.; Long, J. R. Cooperative Carbon Capture and Steam Regeneration with Tetraamine-Appended Metal–Organic Frameworks. *Science* **2020**, *369* (6502), 392–396. https://doi.org/10.1126/science.abb3976.

(25) Philip, F. A.; Henni, A. Incorporation of Amino Acid-Functionalized Ionic Liquids into Highly Porous MOF-177 to Improve the Post-Combustion CO_2 Capture Capacity. *Molecules* **2023**, *28* (20), 7185. https://doi.org/10.3390/molecules28207185.
(26) Lin, M.; Xie, W.; Cheng, X.; Yang, Y.; Sonamuthu, J.; Zhou, Y.; Yang, X.; Cai, Y. Fabrication of Silk Fibroin Film Enhanced by Acid Hydrolyzed Silk Fibroin Nanowhiskers to Improve Bacterial Inhibition and Biocompatibility Efficacy. *Journal of Biomaterials Science, Polymer Edition* **2022**, *33* (10), 1308–1323. https://doi.org/10.1080/09205063.2022.2051694.
(27) Harris, R. K.; Becker, E. D.; Menezes, S. M. C. de; Granger, P.; Hoffman, R. E.; Zilm, K. W. Further Conventions for NMR Shielding and Chemical Shifts (IUPAC Recommendations 2008). *Pure and Applied Chemistry* **2008**, *80* (1), 59–84. https://doi.org/10.1351/pac200880010059.
(28) Geminiani, L.; Campione, F. P.; Canevali, C.; Corti, C.; Giussani, B.; Gorla, G.; Luraschi, M.; Recchia, S.; Rampazzi, L. Historical Silk: A Novel Method to Evaluate Degumming with Non-Invasive Infrared Spectroscopy and Spectral Deconvolution. *Materials* **2023**, *16* (5), 1819. https://doi.org/10.3390/ma16051819.
(29) Laity, P. R.; Gilks, S. E.; Holland, C. Rheological Behaviour of Native Silk Feedstocks. *Polymer* **2015**, *67*, 28–39. https://doi.org/10.1016/j.polymer.2015.04.049.
(30) Giubertoni, G.; Caporaletti, F.; Roeters, S. J.; Chatterley, A. S.; Weidner, T.; Laity, P.; Holland, C.; Woutersen, S. In Situ Identification of Secondary Structures in Unpurified Bombyx Mori Silk Fibrils Using Polarized Two-Dimensional Infrared Spectroscopy. *Biomacromolecules* **2022**, *23* (12), 5340–5349. https://doi.org/10.1021/acs.biomac.2c01156.
(31) Koperska, M. A.; Pawcenis, D.; Bagniuk, J.; Zaitz, M. M.; Missori, M.; Łojewski, T.; Łojewska, J. Degradation Markers of Fibroin in Silk through Infrared Spectroscopy. *Polymer Degradation and Stability* **2014**, *105*, 185–196. https://doi.org/10.1016/j.polymdegradstab.2014.04.008.
(32) Ashok Kumar, R.; Ezhil Vizhi, R.; Sivakumar, N.; Vijayan, N.; Rajan Babu, D. Crystal Growth, Optical and Thermal Studies of Nonlinear Optical γ-Glycine Single Crystal Grown from Lithium Nitrate. *Optik* **2012**, *123* (5), 409–413. https://doi.org/10.1016/j.ijleo.2011.04.019.
